# The Circadian Clock Gene Bmal1 Regulates Microglial Pyroptosis After Spinal Cord Injury via NF‐κB/MMP9

**DOI:** 10.1111/cns.70130

**Published:** 2024-12-08

**Authors:** Dachuan Li, Siyang Liu, Xiao Lu, Zhaoyang Gong, Hongli Wang, Xinlei Xia, Feizhou Lu, Jianyuan Jiang, Yuxuan Zhang, Guangyu Xu, Fei Zou, Xiaosheng Ma

**Affiliations:** ^1^ Department of Orthopedics, Huashan Hospital Fudan University Shanghai China

**Keywords:** Bmal1, MMP9, NF‐κB, pyroptosis, spinal cord injury

## Abstract

**Background:**

The treatment of spinal cord injury (SCI) is usually ineffective, because neuroinflammatory secondary injury is an important cause of the continuous development of spinal cord injury, and microglial pyroptosis is an important step of neuroinflammation. Recently, Bmal1, a core component of circadian clock genes (CCGs), has been shown to play a regulatory role in various tissues and cells. However, it is still unclear whether Bmal1 regulates microglial pyroptosis after SCI.

**Methods:**

In this study, we established an in vivo mouse model of SCI using Bmal1 knockout (KO) mice and wild‐type (WT) mice, and lipopolysaccharide (LPS)‐induced pyroptosis in BV2 cells as an in vitro model. A series of molecular and histological methods were used to detect the level of pyroptosis and explore the regulatory mechanism in vivo and in vitro respectively.

**Results:**

Both in vitro and in vivo results showed that Bmal1 inhibited NLRP3 inflammasome activation and microglial pyroptosis after SCI. Further analysis showed that Bmal1 inhibited pyroptosis‐related proteins (NLRP3, Caspase‐1, ASC, GSDMD‐N) and reduced the release of IL‐18 and IL‐1β by inhibiting the NF‐κB /MMP9 pathway. It was important that NF‐κB was identified as a transcription factor that promotes the expression of MMP9, which in turn regulates microglial pyroptosis after SCI.

**Conclusions:**

Our study initially identified that Bmal1 regulates the NF‐κB /MMP9 pathway to reduce microglial pyroptosis and thereby reduce secondary spinal cord injury, providing a new promising therapeutic target for SCI.

## Graphical abstract

Bmal1 regulates the pyroptosis pathway after spinal cord injury. Bmal1 inhibits the NF‐κB/MMP9 signaling pathway, reduces inflammatory response, and ultimately inhibits pyroptosis in mice spinal cord injury models.

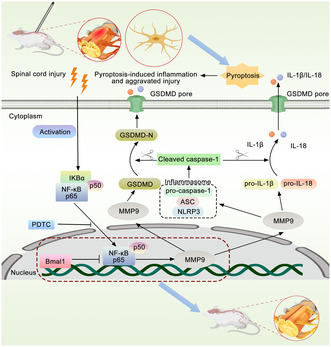

**Abbreviations**
ASC: apoptosis‐associated speck‐like protein containing CARD
CCGs: circadian clock genes
ChIP: chromatin immunoprecipitation
CNS: central nervous system
GSDMD: gasdermin‐D
KO: knockout
LPS: lipopolysaccharide
MMP9: matrix metallopeptidase 9
NF‐κB: nuclear transcription factor‐κB
NLRP3: nucleotide‐binding oligomerization domain‐like receptor thermal protein domain associated protein 3
SCI: spinal cord injury
WT: wild‐type

## 1 Introduction

Spinal cord injury (SCI) is a highly disabling disease in spinal column trauma that causes severe dysfunction below the injured level and affects the quality of life of millions of patients worldwide [1]. In general, the pathological process of SCI mainly includes primary and secondary injury [2]. Primary spinal cord injury denotes the structural damage induced by violent mechanical factors directly acting on the spinal cord tissue. Secondary spinal cord injury is a further injury (including ischemia, hypoxia, cell death, inflammation, oxidative stress) caused by the secretion of a series of cytokines and chemokines due to the changes in the local microenvironment of spinal cord injury, resulting in a “waterfall” biochemical response that causes more severe damage to the spinal cord than the primary injury [3, 4]. Therefore, the neuroinflammatory response induced after SCI is considered by a wide range of researchers to be the most critical link in the pathogenesis of secondary spinal cord injury [5, 6].

Microglia are macrophages in the spinal cord and important mediators of the innate immune response after central nervous system injury, with roles in antigen presentation, cytokine secretion, and phagocytosis in the pathology of neuroinflammation [7, 8, 9]. Upon spinal cord injury, they undergo swift activation, releasing proinflammatory agents and stimulating the generation of reactive oxygen species, culminating in the exacerbation of secondary spinal cord injury. In addition, recent studies on the pathogenesis of various central nervous system diseases have shown that microglia are the main cells in the central nervous system that undergo pyroptosis, and their pyroptosis stands as a significant instigator of neuroinflammation subsequent to spinal cord injury [10, 11]. Therefore, reducing the post‐SCI inflammatory response by inhibiting microglial cell scorching after SCI is crucial for the treatment of SCI and for improving the prognosis of SCI patients.

Pyroptosis is a recently acknowledged form of intensely inflammatory cell demise in recent years. Diverging from conventional apoptosis, pyroptosis is distinguished by the creation of transmembrane pores, cellular swelling, rupture, and the discharge of numerous inflammatory cytokines along with various intracellular components [12]. Activation of nucleotide‐binding oligomerization domain‐like receptors (NLRs) within pyroptotic cells leads to the recruitment of apoptosis‐associated speck‐like protein containing a CARD (ASC) and pro‐caspase‐1, forming inflammasomes. These inflammasomes, in turn, activate caspase‐1, resulting in the cleavage of gasdermin target proteins. Subsequent maturation and release of IL‐1β and IL‐18 were promoted [12, 13, 14]. Therefore, the occurrence of pyroptosis after SCI not only leads to the death of the cell itself but also can further activate and aggravate the inflammatory response in the injured area through the inflammatory factors it releases. However, the regulatory mechanism of pyroptosis in SCI remains to be elucidated.

Bmal1 is a fundamental element of the circadian clock in mammals and it is the primary initiator in the self‐excited positive and negative transcriptional feedback loops formed by biological clock genes which is a key component of central and peripheral rhythm pacemaker sites. The Bmal1 gene is the only clock gene whose knockdown results in complete loss of biorhythmicity in the organism. Recent research has revealed the pivotal role of Bmal1 in governing various neurological conditions, Jung et al. [15, 16, 17] found that Bmal1 gene can reduce the invasiveness of glioma by antagonization of oncogenes. In terms of the immune system, Bmal1 knockout mice exhibited disrupted B cell development, resulting in diminished B cell populations in the bone marrow, spleen, and bloodstream. In addition there is evidence that Bmal1 can exert anti‐inflammatory effects within macrophages, and Chhunchha et al. [18] found that Bmal1 deficiency disrupts NRF2 activity and promotes the accumulation of ROS and pro‐inflammatory factors IL‐1β and IL‐6, thereby promoting an inflammatory response. However, no research have been explored the association between Bmal1 and microglial pyroptosis after SCI.

Therefore, the primary objective of this study was to delve into the involvement of Bmal1 in secondary SCI. and its possible molecular mechanism. Here, we hypothesized that Bmal1 could inhibit microglial pyroptosis, thereby reducing neuroinflammatory responses after SCI. Additionally, we performed a range of in vitro and in vivo experiments to dissect the mechanisms through which Bmal1 governs microglial pyroptosis.

## 2 Materials and Methods

### 2.1 Animals

Bmal1 KO female mice were purchased from the Laboratory Animal Center of the Chinese Academy of Science (Shanghai, China), and WTC57BL female mice were purchased from the Animal Experimental Center of Fudan University. We randomly selected spinal cord tissues from WT mice and Bmal1 KO mice for Western blot to detect the expression of Bmal1, and the results showed that there was a significant difference in Bmal1 content between the two groups, and the knockout efficiency was consistent with the experimental requirements. Moreover, the expression of Bmal1 was measured again after sham operation in the two types of mice, and the results were not significantly different from the control group (Figure S1). To exclude sex differences, all experimental animals were female mice. Mice (9‐week‐old 20–25 g) were resided in standard cages with four mice per cage within a laboratory facility at Fudan University that adhered to specific pathogen‐free conditions. Under conditions of a 12‐h light/dark cycle, regulated temperature, and humidity, the mice were granted a one‐week acclimation period before the commencement of the experiments. All procedures followed in the experiments adhered to the approved experimental protocol set forth by the Animal Protection and Utilization Committee of Fudan University (no. 202203014S).

### 2.2 Spinal Cord Injury and Drugs Treatment

To induce SCI, we performed a surgical procedure targeting the mid‐thoracic region (T8–T9) in mice, as described previously [19]. Briefly, the animals were anesthetized with an intraperitoneal injection of a combination of 70 mg/kg ketamine (Hengrui, China) and 5 mg/kg xylazine (McLean, China) [20]. Each mouse was positioned in a prone orientation on the surgical table, ensuring full exposure of the spine. Subsequently, laminectomy was performed at the T8/T9 lamina. The lateral spinal cord was then compressed to a depth of 0.2 mm for 20 s, thereby inducing a spinal cord contusion. The same surgical procedure, excluding the spinal cord injury, was conducted in the sham group, and skin closure followed after achieving hemostasis. To further investigate the effect of NF‐κB on SCI, after the establishment of SCI model in mice, we referenced and improved the dosage and injection method of Hu et al. [21], the specific operation was as follows. According to the needs of our particular experiment, SCI + NF‐κB inhibitor group was microinjected in NanoFil microinjection system (Sarasota, FL), and the NF‐κB inhibitor pyrrolidine dithiocarbamate (PDTC 30 mg/kg in DMSO, MCE, China) 20 μL was slowly microinjected to a depth of ~1 mm at the site of spinal cord injury. Both the Sham group and SCI group received equal volumes of DMSO (20 μL). Throughout the duration of the experiment, the mice underwent daily monitoring for overall health, cage activity, and signs of infection. The antibiotic gentamicin sulfate (4 mg/kg, Huachu) and meloxicam (1 mg/kg, Boehringer) were administered to the mice during their postoperative recovery for a three‐day period. Manual bladder massage was conducted twice daily until the mice regained the ability to urinate independently.

### 2.3 Evaluation of the Functional Recovery of Mice With SCI

The motor function was evaluated according to Basso Mouse Scale (BMS) to access the motor recovery of mice in each group. The scale is based on observations of the stability of mouse hind limbs in the open field and consists of a 9‐point scale. The mice were given the opportunity to roam freely within a 90‐cm enclosure for a duration of 5 min, during which we closely monitored their hindlimb mobility and coordination [21]. The Sham group, SCI‐WT group, and SCI‐Bmal1 KO group underwent evaluations on postoperative days 1, 3, 7, 14, 21, and 28. The evaluators were blinded to the experimental conditions of the mice, and the assessments were performed three times, with results immediately recorded. The scoring system ranged from 0 to 9, with 0 indicating no motor function and 9 signifying normal function. Footprints were recorded to assess limb coordination by applying red ink to the hind limbs and allowing the mice to walk freely on white A2 paper (594 mm × 420 mm).

### 2.4 Flexibility of the Ankle Joint

**FIGURE 1:**
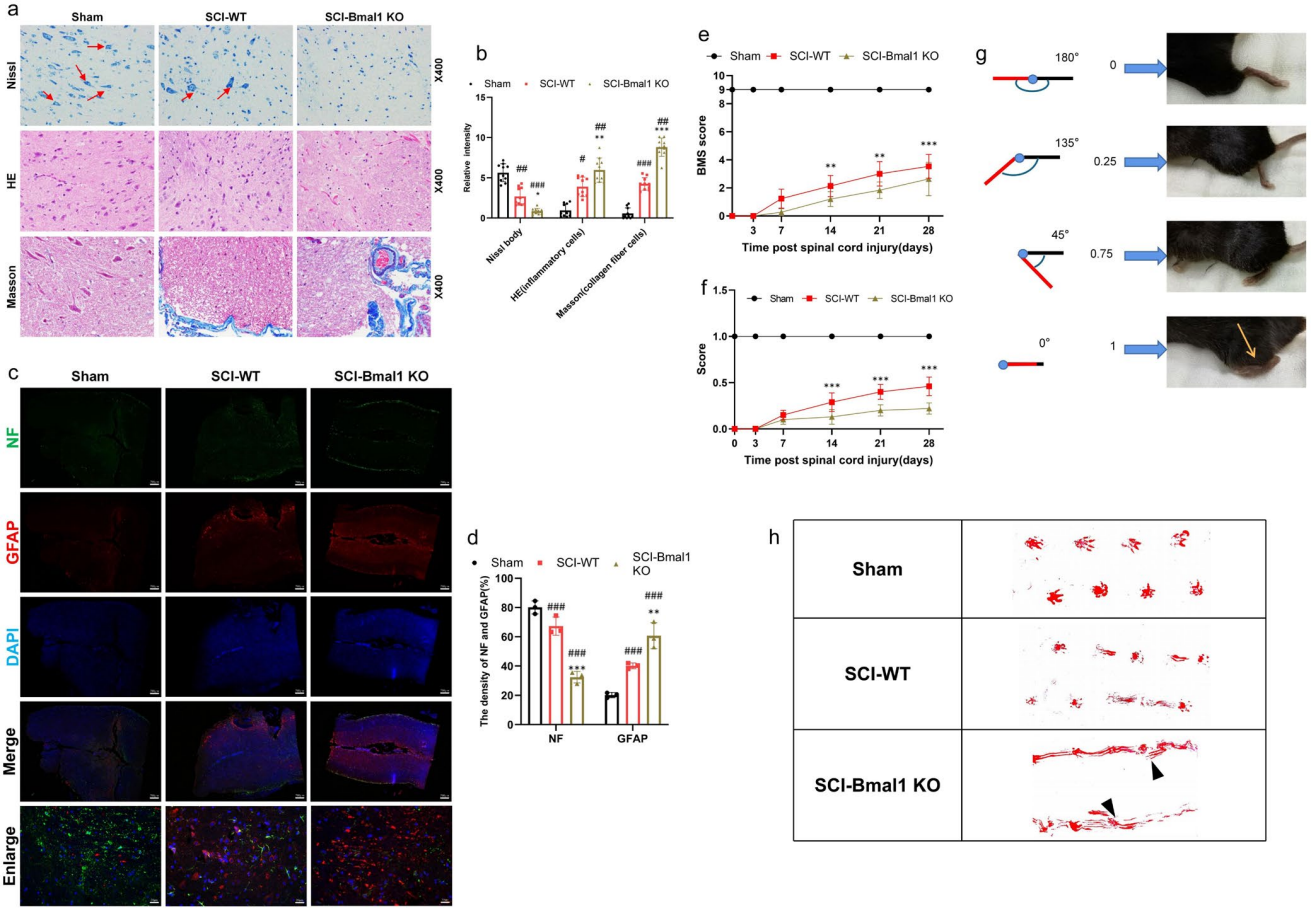
Bmal1 can improve the tissue damage of neurons and promote the recovery of motor function after spinal cord injury in mice. (a, b) Histological analysis of the spinal cords of different groups. Representative spinal cord cross‐sectional HE‐stained images (*n* = 10, scale bar = 50 μm) of the spinal cord of each group of mice 4 weeks after spinal cord injury, with partial magnification of Nissl staining. Nissl staining was utilized to examine the population of motor neurons in the anterior horn of the spinal cord, with the outcomes subjected to analysis. Neurons containing Nissl vesicles were indicated by arrows (*n* = 10, scale bar = 50 μm). And Masson staining of different groups, representing the deposition of collagen in different groups (*n* = 10, scale bar = 50 μm). (c,d) We conducted immunofluorescence staining for NF (red) and GFAP (green). Each group's staining patterns shed light on the functional condition of nerve fibers (*n* = 3, scale bar is 200 and 20 μm). Density of GFAP and NF is assessed in three random fields of view using Image J software. (e) Using BMS scores to assess changes in hindlimb motor recovery during the first 28 days after SCI, assessed at 3, 7, 14, 21, and 28 days (*n* = 10). (f, g) The schematic illustration of the scoring method for assessing ankle flexibility, accompanied by the corresponding ankle flexibility scores for each group (*n* = 10 for each group). (h) Representative images of footprints from each group. The hindlimbs were displayed in red (*n* = 10 for each group). (All the data are expressed as means ± SD, one or two‐way ANOVA followed by Tukey's post hoc test was applied; *p < 0.05, **p < 0.01, ***p < 0.001 vs. SCI‐WT; ^#^ *p* < 0.05, ^##^ *p* < 0.01, ^###^ *p* < 0.001 vs. Sham.)

Muscle spasticity related to ankle dorsiflexion and plantar flexors was assessed during the postoperative analysis of mice [22], and the timing of evaluation coincided with the Basso‐Beattie‐Bresnahan (BBB) scale assessments. The mice were also graded based on various degrees of flexion in their hind limbs, following the criteria as referenced from Dolci et al. [23]. A score of ‘0’ indicated the absence of movement (indicative of spasticity), corresponding to a 180° angle between the tibialis anterior muscle and the paw. A score of ‘0.25’ was assigned for a 135° angle, ‘0.75’ for a 45° angle, and ‘1’ for normal movement, corresponding to a 0° angle (Figure 1).

### 2.5 Cell Culture

BV2 cells were purchased from China iCell and cultured in DMEM (Gibco, Carlsbad, CA) supplemented with 10% fetal bovine serum, 50 μg/mL streptomycin (Invitrogen, Carlsbad, CA), and 50 U/mL penicillin. Cultures were incubated at 37°C and under 95% air and 5% CO_2_ conditions. The pyroptosis of microglia was simulated. OE‐Bmal1, OE‐NF‐κB p65, si‐Bmal1, si‐MMP9 plasmid was constructed by Genechem Ltd., Shanghai, China, and introduced into BV2 cells through transfection using Lipofectamine 2000 reagent (Invitrogen, Carlsbad CA) according to the procedure specified by the manufacturer. Different combinations of lipopolysaccharide (LPS) (1 μg/mL) and PDTC (100 mmol/L) were added 24 h after plasmid formation, and cells were treated for 24 h before subsequent analysis. LPS used in this experiment was purchased from Sigma‐Aldrich (St. Louis, MO, USA), and PDTC from Selleck (NF‐κB inhibitor, S3633; Selleck, USA).

### 2.6 Quantitative Real‐Time PCR (RT‐PCR)

RNA extraction from BV2 cells was carried out utilizing TRIpure reagent (EP013; ELK Biotechnology), following the guidelines provided by the manufacturer. Subsequently, qPCR was conducted using the QuFast SYBR Green PCR system from Life Technologies. The relative mRNA expression was determined through the comparative ΔΔCT method, with GAPDH serving as the reference gene. The primer sequences used in this study are detailed in Table 1.

**TABLE 1:** Sequences of primers used for quantitative real‐time PCR in this study.

Target	Sequence (5′–3′)
*M‐GAPDH*	
Sense	TGAAGGGTGGAGCCAAAAG
Antisense	AGTCTTCTGGGTGGCAGTGAT
*M‐Bmal1*	
Sense	TGCACAATCCACAGCACAG
Antisense	TTCCCATCTATTGCGTGTCG
*M‐MMP9*	
Sense	GTGTCTGGAGATTCGACTTGAAG
Antisense	CAGAAATAGGCTTTGTCTTGGTACT
*M‐NFKBp65*	
Sense	ACTATGAGGCTGACCTCTGCC
Antisense	TCTGGATTCGCTGGCTAATG

### 2.7 Western Blot

BV2 cells were lysed in a buffer containing protease inhibitors (AS1008; Aspen) while kept on ice for 30 min. The protein concentration was determined using the BCA Protein Assay kit (PC0020; Solarbio, Beijing, China). To completely denature the proteins, the protein samples were heated at 100°C for 10 min. Subsequently, the protein samples were separated via SDS‐PAGE electrophoresis and transferred onto PVDF membranes (IPVH00010; Millipore). Following this, the membranes were blocked with 5% skim milk for 1 h and incubated with primary antibodies overnight at 4°C. The antibodies used in this study included anti‐NLRP3 (1:500, ab263899; Abcam), anti‐ASC (1:1000, ab175449; Abcam), anti‐Caspase‐1 (1:500, AF5418; Affbiotech), anti‐Caspase‐11 (1:1000, ab180673; Abcam), anti‐GSDMD (1:1000, #39754; CST), anti‐NFκB p65 (1:1000, #8242; CST), anti‐p‐NFκB p65 (1:500, #3033; CST), anti‐MMP‐9 (1:1000, ab228402; Abcam), anti‐Bmal1 (1:2000, ab230822; CST, Abcam), and anti‐GAPDH (1:10,000, ab181602; Abcam). After three washes with TBST, the membranes were incubated with HRP‐conjugated secondary antibodies for 1 h at room temperature. Finally, imaging was achieved using ECL chemiluminescence substrate (BL520B; Biosharp, China), and the intensity of the bands was quantified using Image J software.

### 2.8 Histological Analysis and Neural‐Like Cell Assessments

At 4 weeks post‐SCI, mice were deeply anesthetized with ketamine at a dosage of 0.1 mg per gram of body weight. Subsequently, they were euthanized, and spinal cord tissues were collected and fixed in 4% (w/v) paraformaldehyde for 24 h. Frozen sections, 20 μm in thickness, were prepared from these tissues using a cryo‐tissue sectioning machine. After fixation for 48 h, the tissues underwent a series of processing steps: they were rinsed in running water for 4 h, gradually dehydrated, soaked in xylene, and finally embedded in paraffin. Subsequently, paraffin sections, 7 μm thick, were obtained by slicing the paraffin‐embedded tissues with a paraffin slicer. The paraffin sections were immersed in water for 5 min and then stained with hematoxylin (Solarbio, Beijing, China) aqueous solution for 5 min, followed by eosin (Solarbio, Beijing, China) staining for 2 min. These steps allowed for the observation of cellular and extracellular matrix characteristics. Light microscopy was used to assess changes in the number of Nissl bodies within the anterior horn of the spinal cord in sections from the same location. In addition, neurofilament protein (NF) staining and glial fibrillary acidic protein (GFAP) staining were used to reflect neuronal cell number and functional status. Immunofluorescence staining was used to label a doublecortin (DCX) protein, which is mainly expressed in dividing neurons and early dividing progeny, and Masson staining was used to determine neurocollagen deposition, thereby representing glial scars.

### 2.9 Immunohistochemical Assessment

For immunohistochemical examination, deparaffinized sections were treated with 3% H_2_O_2_ for 30 min to inhibit endogenous peroxidase activity. Subsequently, they were incubated in a serum‐based blocking solution for an additional 30 min. DAB working solution was prepared by incubation with P65 primary antibody (1:150, AF6387; Affinity) for 1 h, followed by incubation with enzyme‐conjugated anti‐rabbit secondary antibody for 30 min in a water bath at 37°C, and color development was controlled under a microscope. All images were taken using a Nikon ECLIPSE titanium microscope (Nikon, Japan).

### 2.10 Immunofluorescence Assessment

Spinal cord tissue specimens were obtained following the previously outlined procedure. In the case of BV2 cell samples post‐culture treatment, these cells were fixed with 4% paraformaldehyde for 20 min, then subjected to three consecutive 5‐min washes with PBS. Subsequently, the cells were immersed in 0.5% Triton X‐100 for 20 min and subsequently blocked with 1% bovine serum albumin for 60 min. Next, the cells were exposed to primary antibodies overnight at 4°C. Following this incubation, the cells were washed thrice with PBS and incubated with the corresponding secondary antibodies for 40 min at 37°C. All images were captured using a Nikon ECLIPSE Ci‐L microscope (Nikon, Japan), and fluorescence intensity was quantified using Image J software.

### 2.11 Enzyme‐Linked Immunosorbent Assay

The levels of cytokines, including IL‐1β (ELK Biotechnology, ELK1271), IL‐18 (ELK Biotechnology, ELK2269), and TNF‐α (ELK Biotechnology, ELK1387), IL‐4 (ELK Biotechnology, ELK1325), and IL‐10 (ELK Biotechnology, ELK1432) were measured in cell culture supernatants and mouse spinal cord tissue. These measurements were conducted using ELISA kits in accordance with the manufacturer's instructions.

### 2.12 Cytotoxicity Assay

To assess cytotoxicity, the release of lactate dehydrogenase (LDH) was quantified using the LDH Cytotoxicity Assay Kit from Beyotime, Shanghai, China, following the provided instructions by the manufacturer.

### 2.13 Chromatin Immunoprecipitation and Immunoprecipitation

In chromatin immunoprecipitation assays, BV2 cells underwent a 10‐min treatment with 1% formaldehyde to facilitate DNA cross‐linking. Following the cessation of the cross‐linking process, the cell samples were subjected to three successive washes with PBS. Subsequently, the washed cell samples were resuspended in a 10 mM EDTA lysis buffer, adjusted to pH 8.0. Thereafter, the chromatin was broken to 150–250 bp length by disrupting the cells by sonication. The sonicated chromatin solution was diluted 10‐fold with ChIP dilution buffer. To reduce the non‐specific binding Protein background, 20 μL Protein A/G Agarose Beads was added to 2 mL of the sonicated solution. After centrifugation, immunoprecipitated antibodies (IgG was used as a negative control) were added and incubated overnight at 4°C. Immune complexes (antibody–protein–DNA) were harvested and washed once with 1 mL of low salt, 1 mL of high salt and 1 mL of LiCl, respectively. After elution, reverse cross‐linking and DNA purification, qPCR and agarose gel electrophoresis were used for analysis. For immunoprecipitation, the protein content of the samples was quantified utilizing the BCA Protein Concentration Assay kit. The same amount of cell lysate and the corresponding antibody were added to the sample protein, then incubated at 4°C for 1 h, and the appropriate volume of Washing buffer was added for 3 min. Put it on the magnetic frame for 5–10 s. After sucking off the supernatant and collecting the precipitate, after boiling and cooling, resuspended in SDS‐PAGE loading buffer. Protein bands were visualized using specific antibodies in a Western blot analysis.

### 2.14 Luciferase Reporter

Briefly, BV2 cells were plated in 96‐well plates and transfected with a luciferase reporter plasmid, allowing for a 48‐h incubation period. Cells were then lysed and luciferase activity was measured using the veritas 9100‐002 tool (Turner BioSystems, Sunnyvale, CA) according to the manufacturer's instructions.

### 2.15 Transmission Electron Microscopy

BV2 cells were initially fixed with a 2% glutaraldehyde solution for 2 h and subsequently transferred to a 1% citric acid solution for additional fixation. Following fixation, they were immersed in a uranyl acetate solution and gradually dehydrated using a gradient of acetone. The samples were then embedded in epoxy resin, sectioned at a thickness of 70–90 nm, and placed on copper grid troughs. These sections were counterstained with lead citrate, and the ultrastructural features of mitochondria were examined using transmission electron microscopy (TEM).

### 2.16 Statistical Analysis

For all in vitro experiments, *n* = 3 are biological replicates, and the experiments were carried out in at least two independent experiments. SPSS 22.0 (IBM, NY, USA) statistical software was used for analysis. The measurement data are expressed as the means ± standard deviation. When the normal distribution was satisfied (Shapiro–Wilk *W* test) and the variance was homogeneous, the data between the two groups were compared by *t*‐test, the data between the multiple groups were compared by single factor analysis of variance (ANOVA). When the data does not follow the normal distribution or the variance is uneven, the data between the two groups were compared by Wilcoxon rank‐sum test, the data between the multiple groups were compared by Kruskal–Wallis test. The LSD test was used for pairwise comparison. The bilateral inspection level was *α* = 0.05. A *p* value of less than 0.05 was considered to be statistically significant. The figures were drawn by Figdraw and GraphPad Prism 9 (GraphPad, San Diego, CA, USA).

## 3 Results

### 3.1 Bmal1 Ameliorates Neuronal Tissue Damage After Spinal Cord Injury and Promotes Recovery of Motor Function in Mice

SCI models were established to directly test the effect of Bmal1. According to the results of Nissl staining, the Bmal1‐KO group had the least number of Nissl bodies compared with the other groups at 4 weeks after SCI (Figure 1a). A pathological assessment was conducted using hematoxylin and eosin (HE) staining to observe the macroscopic tissue structure. The HE staining results revealed that, following spinal cord injury, the Bmal1‐KO group exhibited noticeably more pronounced inflammatory reactions and tissue damage compared to the other groups. In this group, inflammatory cells increased significantly, and tissue edema exhibited a considerable increase (Figure 1a). Similarly, Masson staining was used to delineate collagen deposition at the site of SCI, and the Bmal1‐KO group showed the highest amount of stained collagen compared to the other groups. More aggregated collagen fibers were detected in Bmal1‐KO mice than in WT mice (Figure 1a,b). Furthermore, additional immunofluorescence staining targeting nerve cells demonstrated that in the spinal cord of Bmal1 knockout mice, the arrangement of NF‐positive cells was significantly irregular and the density was significantly reduced, whereas the number of GFAP‐positive cells was higher than other groups around the spinal cord injury site (Figure 1c,d). Besides, the regeneration of neurons at the entire injury site is crucial for effective spinal cord injury (SCI) repair. DCX, which represents a protein involved in early neuronal differentiation and regeneration, is considered a specific marker of newly generated neurons [24]. Therefore, we utilized immunohistochemical analysis of DCX to evaluate neuronal and axonal regeneration. Additionally, we co‐labeled with the microglia marker iba‐1. The results revealed an increased expression of iba‐1 near the injury site after spinal cord injury, with further elevation following Bmal1 knockout. Conversely, the expression pattern of DCX was entirely opposite (Figure S3). The impact of Bmal1 on the motor recovery of mice was assessed by monitoring Basso Mouse Scale (BMS) scores at 3, 7, 14, 21, and 28 days following spinal cord injury (SCI). Prior to SCI, all mice had a BMS score of 9, and immediately after SCI, this score dropped to 0, indicating a sudden paralysis of both lower limbs due to acute SCI. Over time, scores gradually improved but consistently remained lower in all groups compared with the sham‐operated group (Figure 1e). In the first week after SCI, the WT group recovered the fastest, and then the recovery rate gradually slowed down. The Bmal1‐KO group had a significantly slower recovery of motor function than the other groups (Figure 1e). At the last assessment, a few mice in the WT group could stand on both lower limbs and walk nearly smoothly, but limp was still present. Muscle spasms represent a common indicator of upper motor neuron injury, a prevalent characteristic of spinal cord injury, and are also a prominent contributor to disability in individuals with diverse central nervous system disorders and traumatic injuries [25]. The evaluation of the mice's ankle joint (as illustrated in Figure 1g and detailed in Section 2.4) revealed a gradual improvement in hind limb spasticity with the passage of time (Figure 1f). However, it was observed that the Bmal1‐KO group exhibited the poorest recovery, almost remaining in a state of spastic paralysis after Bmal1 suppression. Next, we investigated whether the absence of Bmal1 following spinal cord injury had an impact on motor function. As depicted in Figure 1h, footprint analysis demonstrated marked irregularities in imprints, characterized by waveform abnormalities and dragging movements, in Bmal1 knockout mice (indicated by black triangles) after SCI. Although the group of wild‐type mice with spinal cord injury exhibited similar footprint irregularities, there were discernible instances of hind limb movements above the ground. In contrast, the Sham group demonstrated the most coordinated movements and did not display distinctive shuffling signs. Consequently, these findings suggest that the inhibition of Bmal1 notably hindered the recuperation of motor function following SCI and exacerbated the condition. In other words, Bmal1 could improve the neural tissue damage after SCI.

### 3.2 Bmal1 Can Inhibit Microglial Pyroptosis in Mice

**FIGURE 2:**
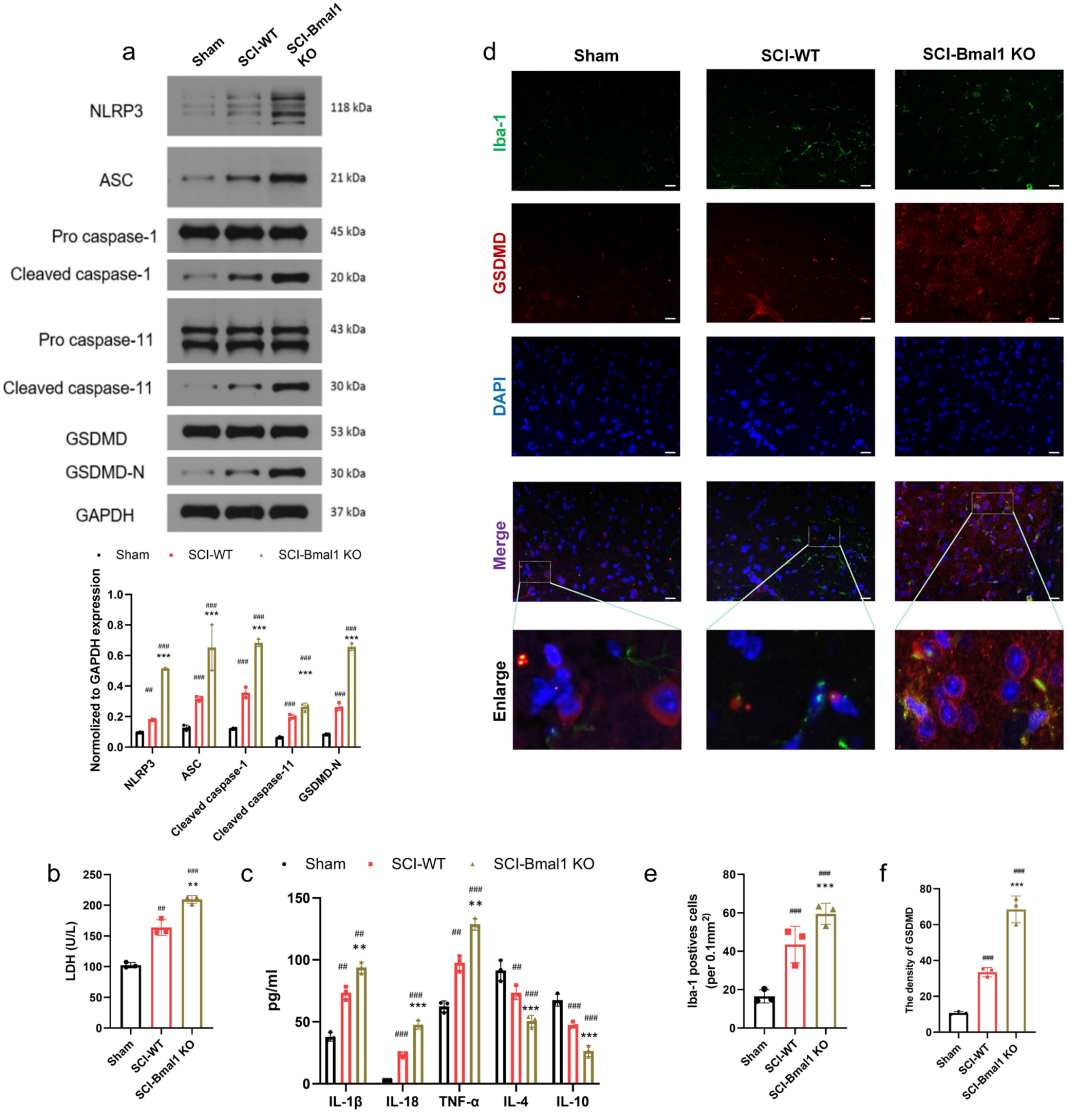
Bmal1 can inhibit microglial pyroptosis in mice. (a) Expression and quantification of pyroptosis‐related molecules (NLRP3, ASC, GSDMD, Cleaved CASP‐1 and Cleaved CASP‐11) after SCI by Western blotting (*n* = 3). (b) The levels of lactate dehydrogenase (LDH) was detected by LDH Assay Kit in different groups (*n* = 3). (c) The levels of inflammatory cytokines (IL‐1β, IL‐18, and TNF‐α) and anti‐inflammatory factors (IL‐4, IL‐10) in different groups measured by ELISA (*n* = 3). (d–f) Representative two‐photon excitation images of immunofluorescence of Iba‐1 and GSDMD acquired from WT or Bmal1 KO mice post‐spinal cord injury or sham surgery as well as quantitative analysis (numbers of Iba1‐positive cells per 0.1 mm^2^). Nuclei are stained with 4′,6‐diamidino‐2‐phenylindole (DAPI) (blue) (*n* = 3, scale bar = 50 μm, the relative contents of lba‐1 and GSDMD were calculated using Image J software). (All the data are expressed as means ± SD, one or two‐way ANOVA followed by Tukey's post hoc test was applied.    **p < 0.01, ***p < 0.001 vs. SCI‐WT; ^##^ *p* < 0.01, ^###^ *p* < 0.001 vs. Sham.)

To further verify the existence of microglial pyroptosis after SCI and the role of Bmal1 in vivo, mice were first subjected to surgery and a series of pyroptosis phenotypes. Western blot results showed that the expression of pyroptosis‐related molecules (NLRP3, ASC, GSDMD, Cleaved CASP‐1 and Cleaved CASP‐11) in the SCI‐Bmal1 KO group was significantly higher than that in the other two groups. Correspondingly, pyroptosis‐related molecules were also significantly increased in the SCI‐WT group compared with the Sham group, but the degree of increase was not as great as that in the Bmal KO group (Figure 2a). Previous studies have shown that activation of NLRP3 inflammasomes in pyroptotic cells requires sufficient levels of NLRP3 protein. Furthermore, the activation of the inflammasome leads to the subsequent activation of the downstream effector, Caspase‐1, which in turn triggers GSDMD‐mediated pyroptosis and the release of proinflammatory cytokines like IL‐1β and IL‐18 [26]. Therefore, Western blot analysis was performed to examine the expression of relevant proteins. These results suggest that pyroptosis is involved in spinal cord injury. The degree of pyroptosis was aggravated after inhibiting Bmal1. Secondly, LDH release data and ELISA assay also demonstrated that the secretion and release of inflammatory cytokines (IL‐1β, IL‐18 and TNF‐α) and LDH were increased after SCI, while inhibiting inflammatory molecules IL‐4 and the expression of IL‐10 is significantly reduced; and the levels of inflammatory cytokines and LDH were higher in Bma1 KO group (Figure 2b,c). In addition, to make the results more robust, immunofluorescence analysis was performed, which also showed that the expression of GSDMD (red fluorescence) in the Bmal1 KO group was significantly increased compared with the other two groups, and it co‐localized with Iba‐1 (microglia marker, green fluorescence). The increased expression of GSDMD was consistent with that of lba‐1. This suggests that pyroptosis occurs predominantly in microglia (Figure 2d). In addition, based on morphological evaluation of multiple magnified microglia cells, we observed a greater trend of microglia soma with increased number of branches and decreased average branch length after SCI and Bmal1 knockdown (Figure S4a,b). These in vivo findings suggest that microglial pyroptosis occurs after SCI, and the decreased expression of Bmal1 upregulates the expression of pyroptosis‐related molecules, inflammatory factors and LDH, suggesting that Bmal1 inhibits microglial pyroptosis after SCI, thereby alleviating spinal cord injury.

### 3.3 Bmal1 Can Inhibit Microglial Pyroptosis After Spinal Cord Injury In Vitro

**FIGURE 3:**
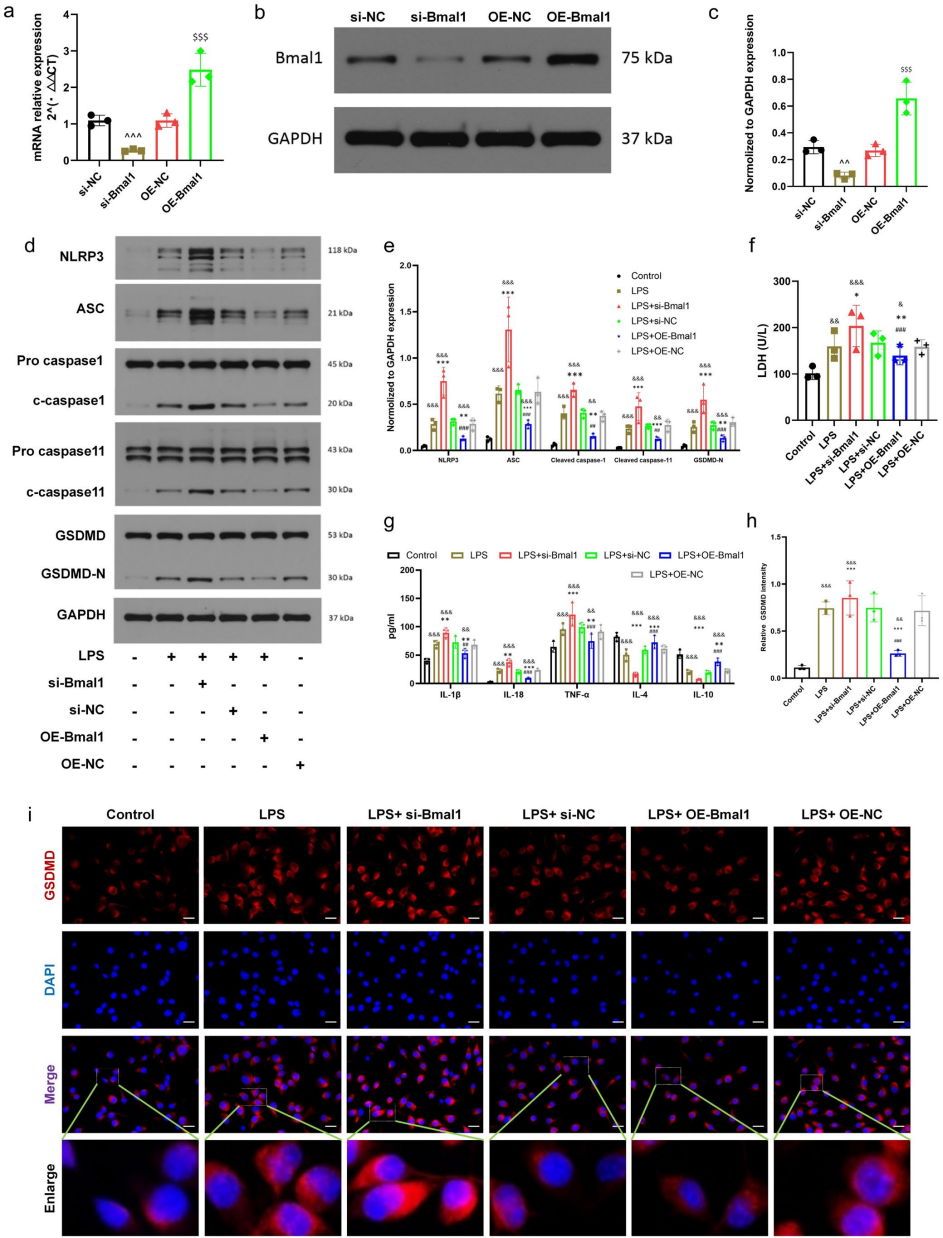
Bmal1 can inhibit microglial pyroptosis after spinal cord injury in vitro. (a–c) Expression of si‐Bmal1 and OE‐Bmal1 in transfected BV2 cells. (d, e) Western blot was used to detect the expression of pyroptosis‐related proteins in BV2 cells of each group and statistical analysis was performed. (f) Lactate dehydrogenase (LDH) release from BV2 cells in each group was detected by lactate dehydrogenase (LDH) detection kit. (g) ELISA was used to detect the release of IL‐1β, IL‐18, and TNF‐α in BV2 cells in different treatment groups. (h, i) Immunofluorescence staining was used to detect the expression level of GSDMD in each group and the quantitative statistical analysis (scale bar = 50 μm, the relative content of GSDMD was calculated by Image J software). (All the data are expressed as means ± SD, *n* = 3, one or two‐way ANOVA followed by Tukey's post hoc test was applied *p < 0.05, **p < 0.01, ***p < 0.001 vs. LPS; ^##^ *p* < 0.01, ^###^ *p* < 0.001 vs. LPS + si‐Bmal1; ^^*p* < 0.01, ^^^*p* < 0.001 vs. si‐NC; ^$$$^ *p* < 0.001 vs. OE‐NC; ^&^ *p* < 0.05, ^&&^ *p* < 0.01, ^&&&^ *p* < 0.001 vs. Control.)

To ascertain if Bmal1 also possesses the capability to suppress pyroptosis in vitro, we used LPS to treat BV2 cells to detect the pyroptosis level and constructed BV2 cells with up—and down‐regulated Bmal1 expression. The PCR and Western blot results demonstrated that the efficacy of Bmal1 knockdown and overexpression met the experimental criteria (Figure 3a–c). In the current WB assay, the overall levels of pyroptosis‐related molecules (NLRP3, ASC, GSDMD‐N, cleaved caspase‐1, and cleaved casepase‐11) increased with the addition of LPS and si‐Bmal1. The expression of pyroptosis‐related molecules was significantly reversed with the up‐regulation of Bmal1 (Figure 3d,e). LDH assay showed that Bmal1 significantly inhibited the release of LDH in BV2 cells (Figure 3f). To further clarify the protective mechanism of Bmal on BV2 cells against inflammatory cytokines, the levels of inflammatory cytokines (IL‐1β, IL‐18, TNF‐α) in each group were tested. LPS + si‐Bmal1 significantly reduced the levels of inflammatory cytokines in the cell culture supernatant (Figure 3g). Immunofluorescence showed similar changes in the expression level of GSDMD in response to up‐regulation and down‐regulation of Bmal1 (Figure 3h,i). Since pyroptosis causes rupture and perforation of the cell membrane, these changes are thought to be the main morphological features that distinguish them from other cell death types [27]. Therefore, we used TEM to observe the effect of Bmal1 on pyroptosis and as shown in Figure S6. Control group and OE‐Bmal1 group showed better cell morphology without typical pyroptosis features. More specifically, we observed that the cells had an intact cell membrane with abundant pseudopodia and that projections were visible around the membrane. On the contrary, we noticed that BV2 cells in LPS group as well as si‐Bmal1 group showed a trend of pyroptosis, among which si‐Bmal1 group showed the most severe pyroptosis, including severe cytoplasmic edema, membrane pore formation and cell membrane disruption (red arrow), global electron density reduction of the cell matrix, and severe cavitation. We quantified the number of pyroptosome observed by electron microscopy (orange triangle) and found that the si‐Bmal1 group had the highest number of pyroptosome. These results collectively suggest that Bmal1 inhibits microglial pyroptosis in vitro.

### 3.4 Bmal1 Can Effectively Inhibit NF‐κB In Vitro and In Vivo

**FIGURE 4:**
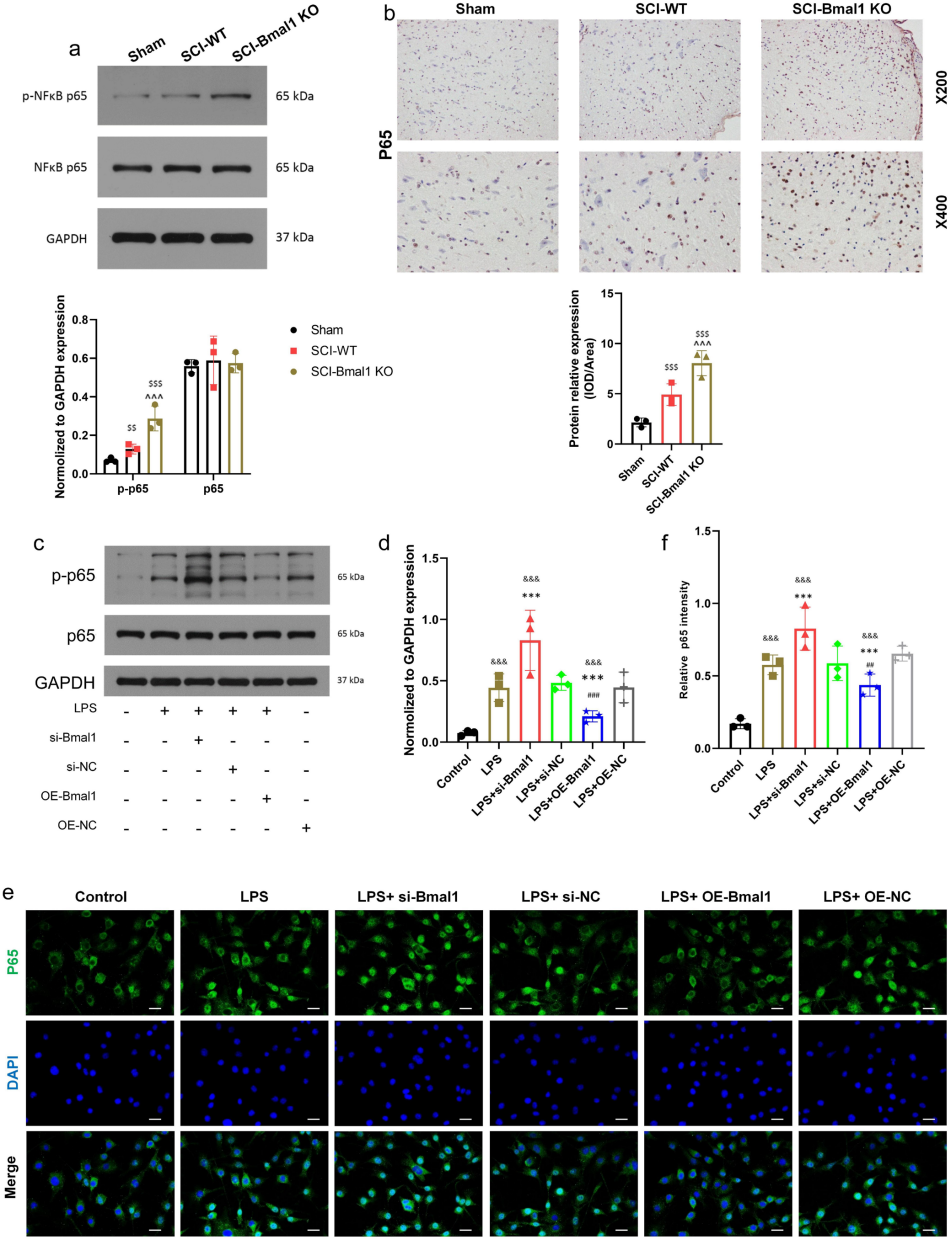
Bmal1 can effectively inhibit NF‐κB in vitro and in vivo. (a) The expression of p‐NF‐κB p65 in the spinal cord of mice was detected by Western blot and quantitative statistical analysis (*n* = 3). (b) The expression of p‐NF‐κB p65 in the spinal cord of mice in each group was detected by immunohistochemistry and statistical analysis (*n* = 3, scale bar = 50 μm, the relative content of p‐NF‐κB p65 was calculated by Image J software). (c, d) Western blot was used to detect the expression of p‐NF‐κB p65 in BV2 cells after up‐regulation or down‐regulation of Bmal1 and quantitative analysis (*n* = 3). (e, f) Immunofluorescence was used to detect the expression of p‐NF‐κB p65 in BV2 cells after up‐regulation and down‐regulation of Bmal1 and quantitative statistical analysis (*n* = 3, scale bar = 50 μm, the relative content of p‐NF‐κB p65 was calculated by Image J software). (All the data are expressed as means ± SD, one‐way ANOVA followed by Tukey's post hoc test was applied ^^^^^ *p* < 0.001 vs. SCI‐WT; ^$$^ *p* < 0.01, ^$$$^ *p* < 0.001 vs. Sham; ^&&&^ *p* < 0.001 vs. Control; ***p < 0.001 vs. LPS; ^##^ *p* < 0.01, ^###^ *p* < 0.001 vs. LPS + si‐Bmal1.)

To further investigate the mechanism through which Bmal1 suppresses microglial pyroptosis, previous research has suggested the possibility of a regulatory connection between Bmal1 and NF‐κB in various tissues and cell types [28]. To determine the role of Bmal1 in NF‐κB regulation after SCI, spinal cord segments from Sham, SCI, and SCI‐Bmal1‐KO mice were collected after surgery. WB and immunohistochemistry results confirmed that the expression of p‐NF‐κB p65 was significantly increased after spinal cord injury and Bmal1 knockout in mice (Figure 4a,b). Secondly, BV2 cells were used to determine the consequences of increasing (overexpression) or decreasing (via siRNA) Bmal1 expression on NF‐kB p65 subunit and the phosphorylated form (p‐p65) in vitro. Western blot and immunofluorescence staining were used to detect the expression of p‐NF‐κB p65 in BV2 cells. As shown in Figure 4c–f, the expression of p‐NF‐κB p65 in BV2 cells of LPS + si‐Bmal1 group was significantly up‐regulated, and the fluorescence intensity was increased. In contrast, upregulation of Bmal1 reversed this effect. This indicated that Bmal1 inhibited the expression of NF‐κB. In addition, previous studies have suggested that NF‐κB is an essential and ubiquitous transcription factor for the activation of various proinflammatory mediators such as TNF‐α and IL‐1β in microglia [29] therefore, we found by immunofluorescence that under untreated conditions, the NF‐κB p65 subunit was almost all located in the cytoplasm, and when BV2 cells were exposed to LPS, p65 protein appeared mainly in the nucleus, confirming the activation of inflammatory response in microglia and the induction of nuclear translocation of NF‐κB p65 (Figure 4e). Taken together, these results collectively suggest that Bmal1 attenuates microglial pyroptosis by inhibiting NF‐κB expression.

### 3.5 NF‐κB Is a Transcriptional Activator in the Promoter Region of MMP9 Gene, Which Promotes Microglial Pyroptosis by Regulating MMP9

**FIGURE 5:**
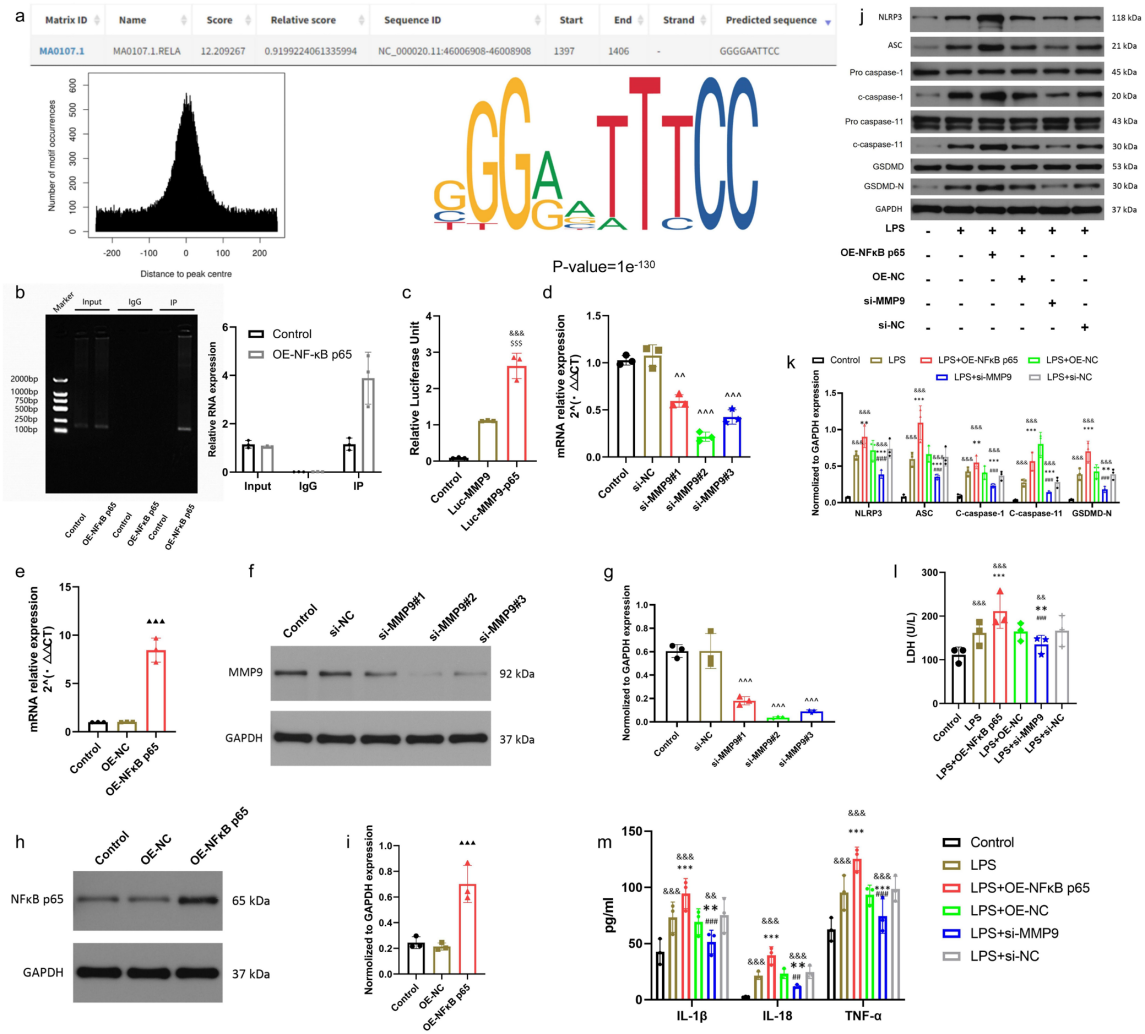
NF‐κB is a transcriptional activator in the promoter region of MMP9 gene, which promotes microglial pyroptosis by regulating MMP9. (a) JASPAR database predicts NF‐κB binding sites in the MMP9 promoter region. (b) ChIP results showed that NF‐κB could directly bind to MMP9. (c) Dual‐luciferase reporter assay for NF‐κB and MMP9. (d, e) PCR was used to detect the expression of NF‐κB p65 and MMP9 in BV2 cells after up‐regulation and down‐regulation. (f, g) Western blot and quantitative analysis of si‐MMP9 expression. (h, i) Western blot and quantitative analysis of NF‐κB p65 overexpression. (j, k) Western blot was used to detect the expression of pyroptosis‐related molecules in BV2 cells and quantitative statistical analysis was performed. (l) Lactate dehydrogenase (LDH) release from BV2 cells in each group was detected by lactate dehydrogenase (LDH) detection kit. (m) ELISA was used to detect the release of IL‐1β, IL‐18 and TNF‐α in BV2 cells in different treatment groups. (All the data are expressed as means ± SD, *n* = 3, one‐way or two‐way ANOVA followed by Tukey's post hoc test was applied. **p < 0.01, ***p < 0.001 vs. LPS; ^##^ *p* < 0.01, ^###^ *p* < 0.001 vs. LPS + si‐Bmal1; ^^^^ *p* < 0.01, ^^^^^ *p* < 0.001 vs. si‐NC;   ^$$$^ *p* < 0.001 vs. Luc‐MMP9; ^▲▲▲^ *p* < 0.001 vs. OE‐NC; ^&&^ *p* < 0.01, ^&&&^ *p* < 0.001 vs. Control.)

Numerous investigations have indicated that NF‐κB can modulate the expression of MMP9 [30, 31]. Given the NF‐κB is one of the important transcription factors involved in various diseases, to explore the molecular regulation of relationship between them, therefore, in this study, we first use the JASPAR database (http://jaspar.genereg.net) identification of MMP9 promoter region with potential binding sites of the NF‐κB. The results also indicated a potential binding site between NF‐κB and MMP9 promoter region. NF‐κB may act as a transcriptional activator to promote MMP9 expression (Figure 5a). ChIP results showed a direct binding of NF‐κB to the MMP9 promoter region, further confirming this speculation (Figure 5b). Luciferase assay showed consistent with ChIP results, indicating the presence of NF‐κB binding sites in the MMP9 gene promoter region in BV2 cells (Figure 5c). In addition, to continue to analyze the molecular mechanism of the two involved in microglial pyroptosis, downregulation of MMP9 or up‐regulation of NF‐κB p65 in BV2 cells was performed to determine the regulatory effect. The results of PCR showed that the knockdown and overexpression efficiency of MMP9 and NF‐κB p65 met the experimental requirements (Figure 5d,e). WB and its quantitative results further indicated that our part was feasible for the down‐regulation of MMP9 (Figure 5f,g). Furthermore, the effect of overexpression of NF‐κB p65 also met the needs of this experiment (Figure 5h,i). As expected, Western blot results showed that overexpression of NF‐κB p65 and downregulation of MMP9 significantly promoted and inhibited the expression of pyroptosis‐related molecules in BV2 cells, respectively, confirming their common promoting effect on pyroptosis from both positive and negative aspects, The expression of pyroptosis‐related proteins(NLRP3, ASC, GSDMD, Cleaved caspase‐1 and Cleaved caspase‐11) was significantly down‐regulated after the overexpression of NF‐κB p65, while it was significantly increased after the overexpression of si‐MMP9 (Figure 5j,k). Consistently, LDH and ELISA assays showed that plasma‐treated BV2 cells significantly increased the release of inflammatory cytokines IL‐1β, IL‐18, TNF‐α, and LDH. Specifically, knockdown of MMP9 aggravated the inflammatory response, while overexpression of NF‐κB p65 slowed the release of inflammatory factors and LDH (Figure 5l,m). Therefore, we suggest that NF‐κB promotes microglial pyroptosis by mediating MMP9 expression at the transcriptional level. These results open the possibility for us to discuss the mechanism further.

### 3.6 Bmal1 Inhibits LPS‐Induced Pyroptosis Mediated by NF‐κB/MMP9 Signaling Pathway in BV2 Cells

**FIGURE 6:**
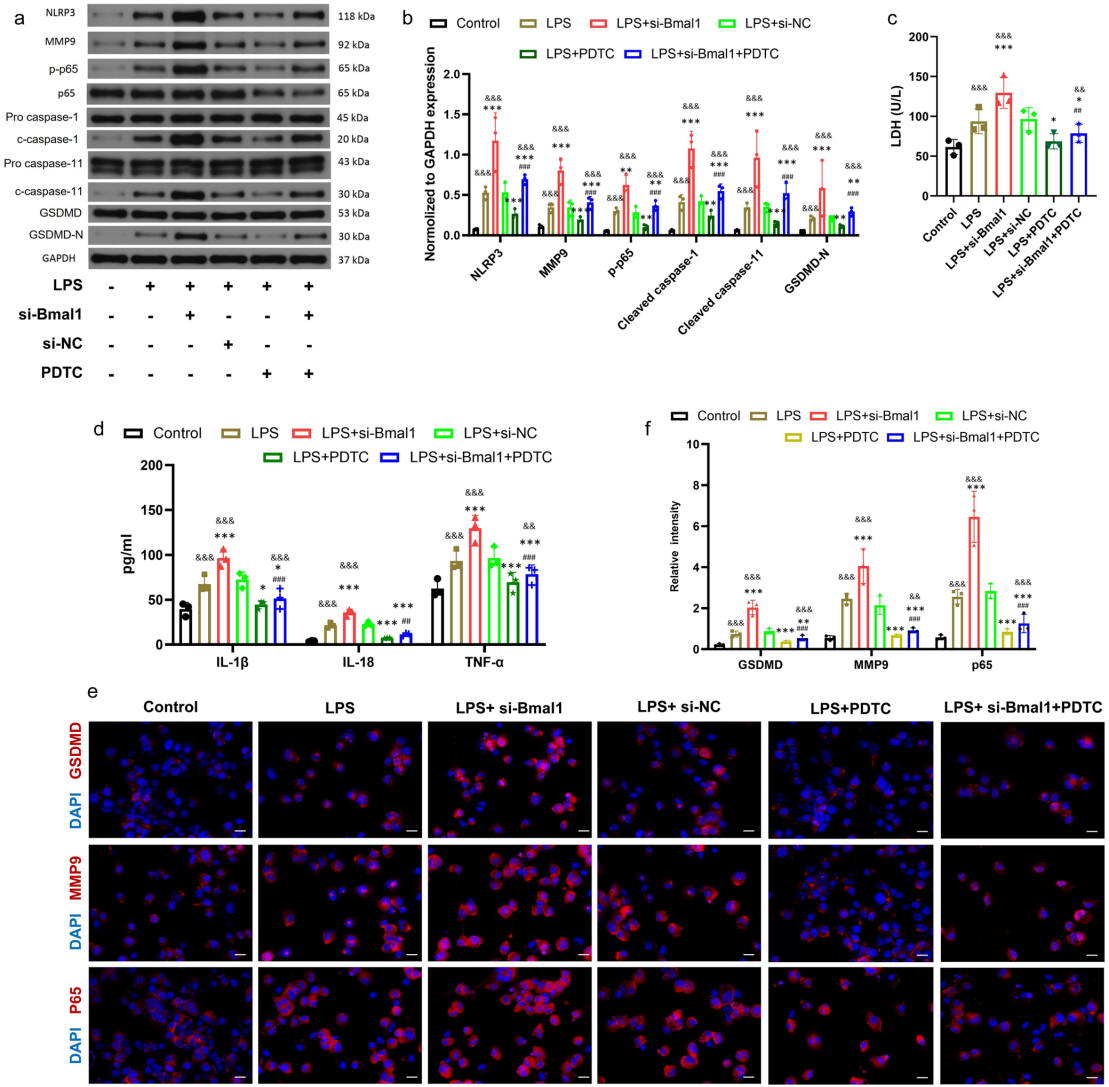
Bmal1 inhibits LPS‐induced pyroptosis mediated by NF‐κB/MMP9 signaling pathway in BV2 cells. (a, b) Western blot was used to detect and quantify the expression of pyroptosis‐related proteins, p‐p65 and MMP9 under Bmal1 knockdown and PDTC treatment. (c, d) The release of LDH and the secretion of inflammatory factors in each group after knocking down Bmal1 and PDTC treatment were detected and quantified. (e, f) Immunofluorescence staining was used to detect the expression of GSDMD, MMP9 and p65 in each group and quantitative statistical analysis was performed (scale bar = 50 μm, the relative content of GSDMD, MMP9 and p65 was calculated by Image J software). (All the data are expressed as means ± SD, *n* = 3, one or two‐way ANOVA followed by Tukey's post hoc test was applied *p < 0.05, **p < 0.01, ***p < 0.001 vs. LPS; ^##^ *p* < 0.01, ^###^ *p* < 0.001 vs. LPS + si‐Bmal1; ^&&^ *p* < 0.01, ^&&&^ *p* < 0.001 vs. Control.)

We hypothesized that Bmal1 inhibited microglial pyroptosis by inhibiting the NF‐κB/MMP9 pathway. Next, we determined the regulatory role of Bmal1 on NF‐κB/MMP9 and pyroptosis by down‐regulating Bmal1 and treating cells with NF‐κB inhibitor PDTC (100 mmol/L, 24 h) in vitro. The Western blot outcomes revealed a notable increase in the expression levels of pyroptosis‐related proteins in BV2 cells following Bmal1 knockdown, which was consistent with the previous in vitro results, and the expression levels of p‐65 and MMP9, two important molecules, were also significantly increased. These results suggested that the inhibitory effect of Bmal1 on the two proteins was weakened after knockdown of Bmal1, after PDTC was added, the increased expression of pyroptosis‐related proteins induced by LPS was reversed. However, after Bmal1 knockdown the pyroptosis‐related proteins (NLRP3, ASC, GSDMD, Cleaved caspase‐1 and Cleaved caspase‐11), p‐p65 and MMP9 expression levels were significantly increased. On the contrary, the enhanced pyroptosis and the up‐regulation of p‐p65 and MMP9 induced by Bmal1 knockdown were significantly reversed after PDTC treatment. As hypothesized, NF‐κB regulates the downstream MMP9 expression, and the NF‐κB/MMP9 signaling pathway mediated pyroptosis can be inhibited by Bmal1 (Figure 6a,b). In addition, the results of our LDH and ELISA assays confirmed this phenomenon, inhibition of Bmal1 aggravated the inflammatory response, whereas inhibition of NF‐κB attenuated the release of inflammatory factors and LDH (Figure 6c,d). Immunofluorescence also showed that knockdown of Bmal1 significantly promoted the expression of GSDMD, MMP9 and p65, which were effectively suppressed by inhibition of NF‐κB activity (Figure 6e,f). Collectively, these results indicated that Bmal1 inhibited the NF‐κB/MMP9 axis to suppress pyroptosis in BV2 cells.

### 3.7 Bmal1 Inhibits Microglial Pyroptosis After Spinal Cord Injury In Vivo by Inhibiting NF‐κB/MMP9

**FIGURE 7:**
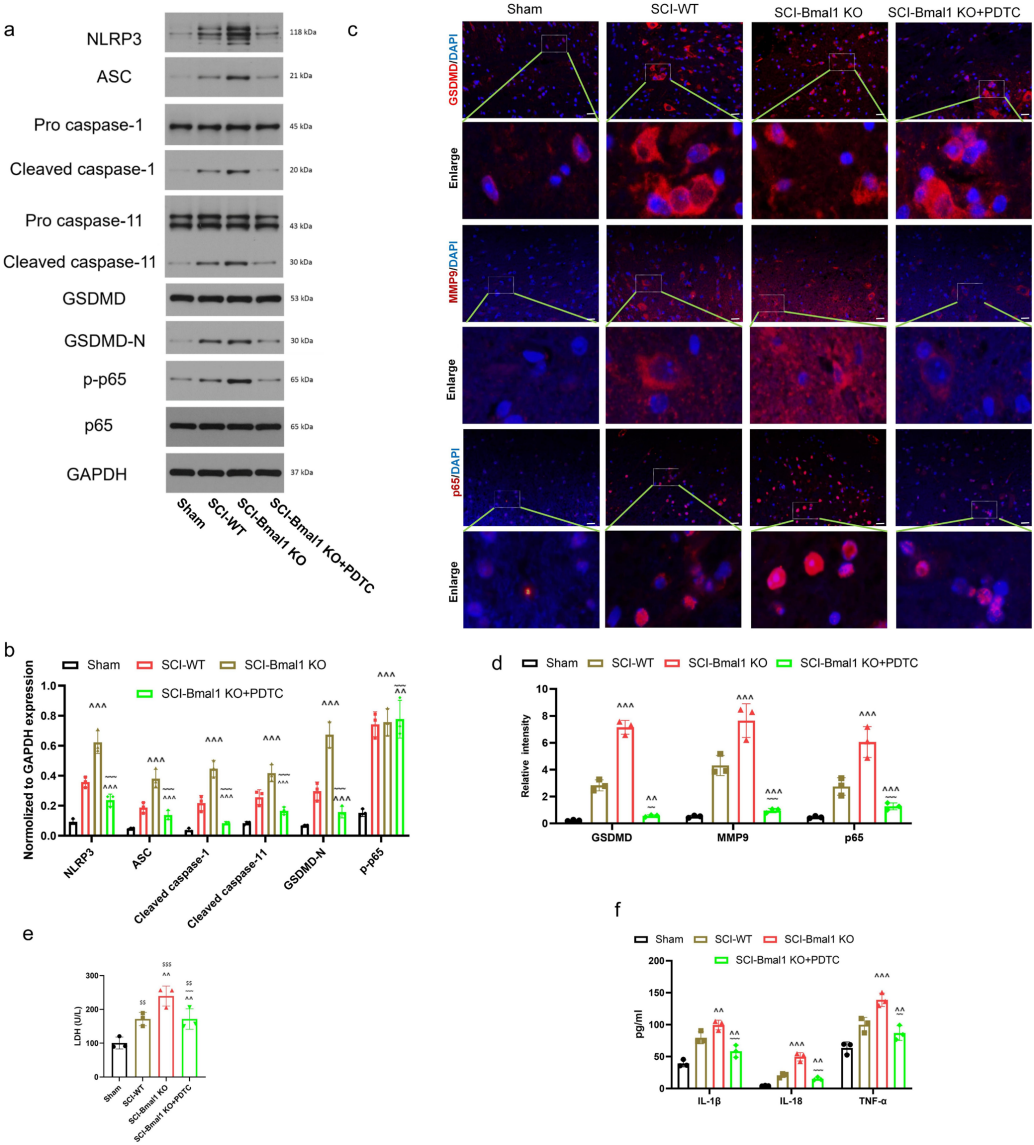
Bmal1 inhibits microglial pyroptosis after spinal cord injury in vivo by inhibiting NF‐κB/MMP9. (a, b) Western blot analysis was used to identify and quantitate the expression of pyroptosis‐related proteins in PDTC cells added (or not added) after SCI (*n* = 3). (c, d) Immunofluorescence staining was utilized for the assessment of GSDMD, MMP9, and p65 expression, followed by subsequent quantitative statistical analysis (scale bar = 50 μm, the relative content of GSDMD, MMP9 and p65 was calculated by Image J software). (e, f) The release of LDH in different groups and the expression levels of inflammatory factors in different groups were detected by ELISA. (All the data are expressed as means ± SD, *n* = 3, one‐way or two‐way ANOVA followed by Tukey's post hoc test was applied ^~~^ *p* < 0.01, ^~~~^ *p* < 0.001 vs. SCI‐Bmal1 KO; ^^^^ *p* < 0.01, ^^^^^ *p* < 0.001 vs. SCI‐WT; ^$$^ *p* < 0.01, ^$$$^ *p* < 0.001 vs. Sham.)

To further clarify that the mechanism by which Bmal1 regulates microglial pyroptosis after SCI is mediated through the NF‐κB/MMP9 signaling pathway, we microinjected some Bmal1‐KO mice with an NF‐κB inhibitor (PDTC 30 mg/kg, 20 μL) at the time of surgery. Thus, the validation is more complete. Next, we examined spinal cord tissue by Western Blot and found that, consistent with previous results, The expression levels of pyroptosis‐related proteins (NLRP3, ASC, GSDMD‐N, cleaved caspase‐1 and cleaved casepase‐11) and p‐NF‐κB p65 protein were increased most significantly in the SCI‐Bmal1 KO group. However, the addition of PDTC could significantly reverse this increase in expression, and the expression level of related proteins was even lower than that of SCI‐WT. As expected, inhibition of NF‐κB significantly suppressed the level of pyroptosis after SCI (Figure 7a,b). The results of immunofluorescence staining were consistent with those of Western Blot, and the staining of MMP9 showed that inhibition of NF‐κB in addition to reducing cell pyroptosis also significantly inhibited the expression of downstream MMP9 (Figure 7c,d). LDH release data and ELISA assay further demonstrated that NF‐κB inhibition reduced cellular inflammation after SCI (Figure 7e,f). Based on these data, NF‐κB/MMP9 signaling is involved in regulating microglial pyroptosis after Bmal1‐inhibited SCI in vivo.

## 4 Discussion

Spinal cord injury (SCI) is a traumatic and severe disorder affecting the nervous system, which brings a huge burden to the patient's body, body and social economy. Neuroinflammation, oxidative stress, etc. are all involved in the damage process, which eventually leads to different cell death subroutines [32]. It involves neuroinflammation, oxidative stress, and various cell death mechanisms, complicating the repair process. Therefore, interfering with cell death pathway is one of the main strategies for repair [33]. Targeting cell death pathways, including apoptosis, autophagy, pyroptosis, necroptosis, and ferroptosis, is essential for effective SCI repair [34, 35, 36, 37]. Pyroptosis is a pro‐inflammatory programmed cell death named by Cookson and Brennan [38]. In terms of genetic, metabolic and protein regulators, pyroptosis distinguishes itself from other types of regulated cell death [39, 40]. Following the proposal of pyroptosis, several researchers discovered morphological, biochemical, and molecular evidence supporting the presence of pyroptosis in SCI. Therefore, blocking pyroptosis may be a promising therapeutic strategy to alleviate spinal cord tissue injury and accelerate neurological function recovery. Since spinal cord injury is often accompanied by inflammation, LPS, as a mature inducer, has been widely used to construct a cell pyroptosis model after spinal cord injury in vitro [41, 42]. In the in vitro experiment, to successfully construct a cell pyroptosis model, we used LPS to treat the cells. According to our results, LPS could significantly increase the expression of pyroptosis‐related proteins in BV2 cells (Figure 3). In the in vivo study, we found similar results (Figure 2). This part of the results is consistent with the current understanding that characterizes pyroptosis [43]. Therefore, pyroptosis unquestionably participates in the pathological course of SCI.

The Bmal1 (clock gene) gene is a core regulator of the molecular clock, driving rhythmic circadian transcription in the nucleus [44]. However, the exact role of Bmal1 in regulating programmed cell death in various inflammatory diseases remains unclear. Recently, Ramsey et al.'s study [45] found that the deletion of Bmal1 can reduce the activity of Nrf2 (nuclear factor E2‐related factor 2) and lead to increased production of inflammatory cytokines, which aggravates the inflammatory response in the brain of mice with cerebral ischemia–reperfusion. In conclusion, Bmal1 may be deeply involved in the pathological process of pyroptosis during secondary inflammation after SCI. Therefore, in our study, we manipulated the expression of Bmal1 in BV2 cells in vitro by both upregulation and downregulation. It was found that knockdown of Bmal1 could significantly promote pyroptosis and inflammatory response in BV2 cells. On the contrary, OE‐Bmal1 inhibited pyroptosis (Figure 3). In the following animal experimental study, the results were as expected. First, the results showed that the pyroptosis of spinal microglia was significantly aggravated after Bmal1 knockout (Figure 2). In addition, the inhibition of Bmal1 worsened the severity of spinal cord injury (Figure 1). These results are similar to Xiang et al. [46] that Bmal1 can alleviate central nervous system injury, and our research provides the initial confirmation. In addition, studies have shown that Bmal1 regulates diurnal variation in monocytes and impacts their trafficking to local sites of inflammation [47], and perhaps the reduction in inflammatory factors in our results is also related to this mechanism. Indeed, Bmal1 possesses the capacity to suppress microglial pyroptosis following spinal cord injury in vitro and in vivo.

However, it has been shown that loss of Bmal1 after SCI improves neurological function, reduces inflammation and protects the blood‐spinal cord barrier. This appears to be contrary to our findings and those of previous studies [48]. We think the possible reason is that: (1) In the study by Slomnicki et al., it was suggested that the repair of spinal cord injury due to Bmal1 deficiency was improved in the blood‐spinal cord barrier by promoting angiogenesis. However, scar formation and neuronal fibrosis after SCI over time may also manifest as increased cell and vascular proliferation. In addition, in the subacute phase (2–4 days after injury), vascular thrombosis leads to further ischemia [49]. (2) The reduction in neuroinflammation observed in the study by Slomnicki et al. may be due to endogenous repair of the injured spinal cord caused by accumulation of antiinflammatory microglia at the site of injury during the acute phase after Bmal1 knockdown. The regulation of inflammation by Bmal1 is mainly manifested through macrophages. Furthermore, Bmal1 may control the rhythm changes of monocytes and other immune cells, and knockout of Bmal1 may cause the rhythm disruption of these cells [47], so the phenotypic changes of microglia after injury should be specifically analyzed, which will also lead to the differences in the conclusions of the two studies [50]. Further experiments are needed to determine the exact reason.

Next, we explored the specific molecular mechanism by which Bmal1 inhibits pyroptosis. The NF‐κB pathway is a major pathway involved in inflammation after acute injury in various systems [51, 52]. It is precisely because of the proinflammatory function of NF‐κB that there is a rationale for linking NF‐κB to pyroptosis after SCI. Previously, Liu's group reported that NF‐κB signaling promotes microglial pyroptosis sensitivity after SCI^42^. Fan et al. [53] found that Bmal1 can effectively down‐regulate NF‐κB activation, which is a negative regulator of NF‐κB signaling. Therefore, we first used Bmal1 knockout mice to verify the expression of NF‐κB p65 at the tissue and molecular levels in vivo, and then used BV2 cells to verify the regulatory relationship between Bmal1 and NF‐κB p65, which was in line with the previous findings. Decreasing Bmal1 expression considerably heightened the p65 expression, while elevating Bmal1 expression effectively reversed this outcome (Figure 4).

At present, many studies on the regulation of pyroptosis by NF‐κB focus on the direct promotion of inflammatory factors and pyroptosis‐related molecules by NF‐κB, thereby regulating inflammation [54, 55]. However, the specific transcriptional regulation mechanism of NF‐κB downstream is rarely reported. Therefore, it is very important to further study the mechanism of NF‐κB mediating pyroptosis. Experiments by Zhou et al. [56] showed that inhibition of MMP9 expression could reduce neuronal pyroptosis after blood‐brain barrier damage. These results suggest that targeting MMP9 may be an important step to reduce pyroptosis. Therefore, the question arises: is there an upstream and downstream regulatory association between NF‐κB and MMP9 that impacts pyroptosis? Kim et al. [30] first found the promoter region of MMP9 has a binding site for nuclear factor NF‐κB, and its expression is regulated by upstream NF‐κB. Therefore, we also hypothesized that MMP9 expression in microglia was regulated by NF‐κB after SCI. Using the JASPAR database (http://jaspar.genereg.net) to predict the NF‐κB indeed for potential transcription factor of MMP9. Then, we used a series of experiments to confirm that MMP9 is an NF‐κB binding site (Figure 5), and NF‐κB may inhibit microglial pyroptosis after SCI by regulating MMP9 expression. Finally, we further tested our hypothesis that the NF‐κB/MMP9 pathway is indispensable for Bmal1 in the regulation of pyroptosis. The in vitro and in vivo experiments were performed to evaluate the effects of Bmal1 knockdown and addition of NF‐κB inhibitor PDTC on the NF‐κB/MMP9 pathway and pyroptosis‐related molecules. As shown in Figures 6 and 7, Bmal1 inhibited the pro‐pyroptosis effect of NF‐κB and MMP9, and the results indicated that inhibition of NF‐κB further enhanced the anti‐pyroptosis effect of Bmal1. Taken together with our previous results, we can conclude that Bmal1 regulates microglial pyroptosis after SCI by inhibiting NF‐κB/MMP9 signaling pathway. According to our study, inhibition of NF‐κB/MMP9 signaling by Bmal1 may be a mechanism by which Bmal1 mediates pyroptosis after SCI.

Although Bmal1's primary function is to mitigate microglial pyroptosis following SCI, the ultimate objective is to enhance functional recovery after SCI. Our experimental outcomes affirm the effectiveness of Bmal1, as evidenced by the improvements in both histological findings and functional behaviors. Furthermore, we found Bmal1 can act on cytokines to alleviate spinal cord injury. To our knowledge, our study represents the inaugural report establishing a connection between Bmal1, SCI, and pyroptosis, and the findings present a promising avenue for therapeutic strategies in the central nervous system. However, the study has some limitations. First, as Bmal1 is a circadian rhythm‐related molecule, it remains uncertain whether Bmal1's impact extends to other metabolism‐related signaling pathways within the central nervous system and whether its regulatory influence operates through alternative metabolic pathways, which still needs to be further investigated after SCI. Second, the more intricate regulatory connection between Bmal1 and NF‐κB warrants further validation. Although earlier research has identified the intermediate factor CLOCK, the precise mechanism remains ambiguous. In addition, contrary to our results, it has been suggested that loss of Bmal1 seems to attenuate the inflammatory response after SCI, which may be related to the repair mechanism after injury, and the nerve cell type used in specific experiments. Therefore, although the current study contributes to the understanding of the mechanism of microglial pyroptosis, further experimental validation is needed. Furthermore, in this experiment, we limited the use of female mice. However, recent studies have shown that there are significant gender differences in gene expression and metabolism in the central nervous system [57, 58]. The effect of gender differences on recovery after SCI in mice has been neglected and needs to be improved in future studies.

## 5 Conclusion

Bmal1 may be considered as a new target for the treatment of SCI. Bmal1 can alleviate spinal cord injury by reducing microglial pyroptosis by regulating the NF‐κB/MMP9 signaling pathway.

## Author Contributions

Dachuan Li and Xiao Lu performed the experiments and wrote the original manuscript. Siyang Liu and Zhaoyang Gong analyzed the data and prepared the figures. Hongli Wang, Xinlei Xia, Feizhou Lu, and Jianyuan Jiang have revised the manuscript. Yuxuan Zhang, Guangyu Xu, Fei Zou, and Xiaosheng Ma conceived and designed the experiments and provided funding. All authors reviewed and approved the final manuscript.

## Ethics Statement

All animal experiments were formally examined and approved the Animal Protection and Utilization Committee of Fudan University (No. 202203014S).

## Consent

The authors have nothing to report.

## Conflicts of Interest

The authors declare no conflicts of interest.

## Supporting information


Appendix S1.

## Data Availability

The data that support the findings of this study are available from the corresponding author upon request.
