# Supplementary material for: The Circadian Clock Gene Bmal1 Regulates Microglial Pyroptosis After Spinal Cord Injury via NF‐κB/MMP9
Source: CNS Neurosci Ther. 2024 Dec 8;30(12):e70130. doi: 10.1111/cns.70130 (PMC11625957; doi:10.1111/cns.70130)
Supplement: Supplementary file 1 — Appendix S1. [file CNS-30-e70130-s001.pdf]

Figure S1

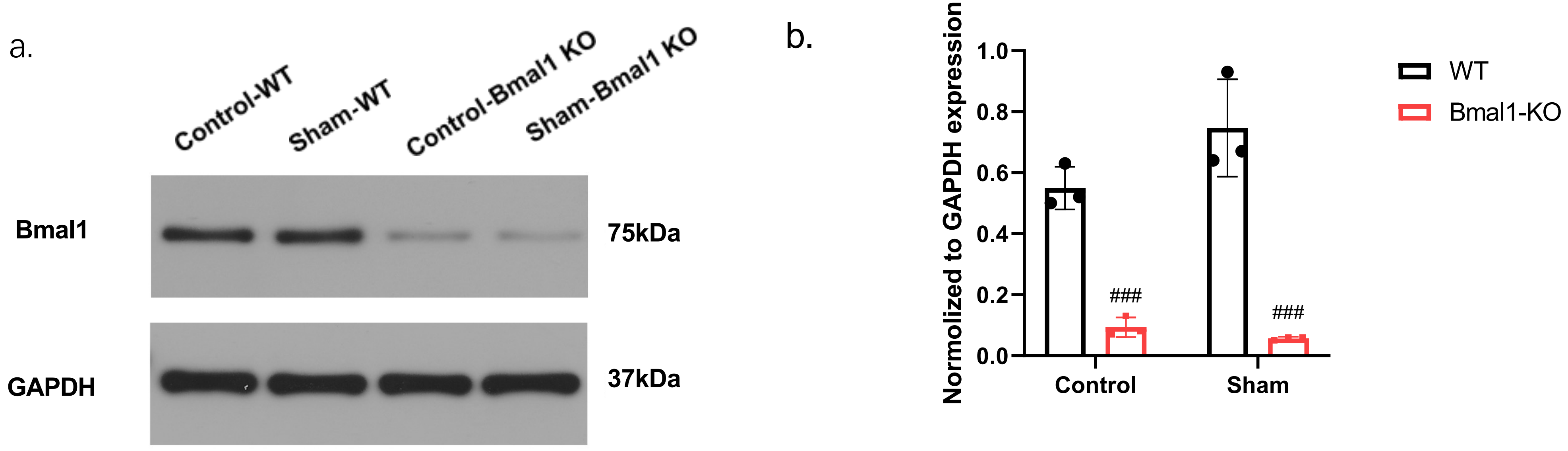

Figure S1． （a-b） Western blot was used to detect the expression of Bmal1 in in WT mice and Bmal1KO mice and statistical analysis was performed. (n=3． All the data are expressed as means ±SD, one -way ANOVA followed by Tukey's post hoc test was applied; # P < 0.05, ## P < 0.01, ### P < 0.001 vs. WT.)

Figure S2

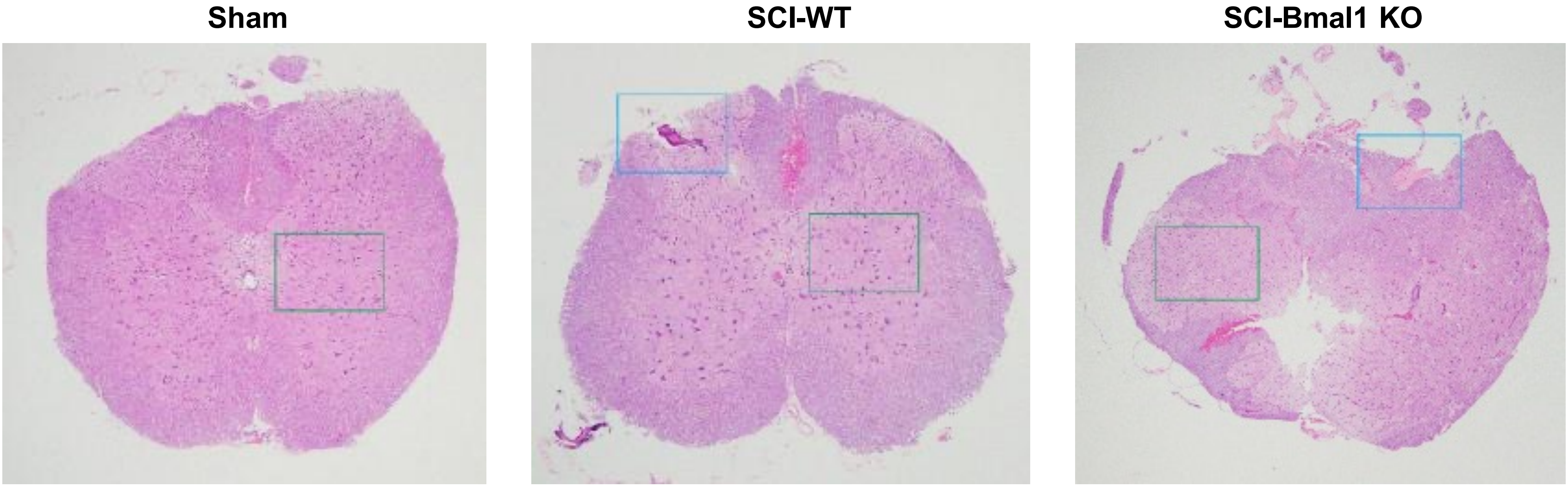

Figure S2. HE-stained images of complete cross-sectional sections of the spinal.(The inflammatory response is indicated in the green box, and scar tissue generation is indicated in the blue box)

Figure S3

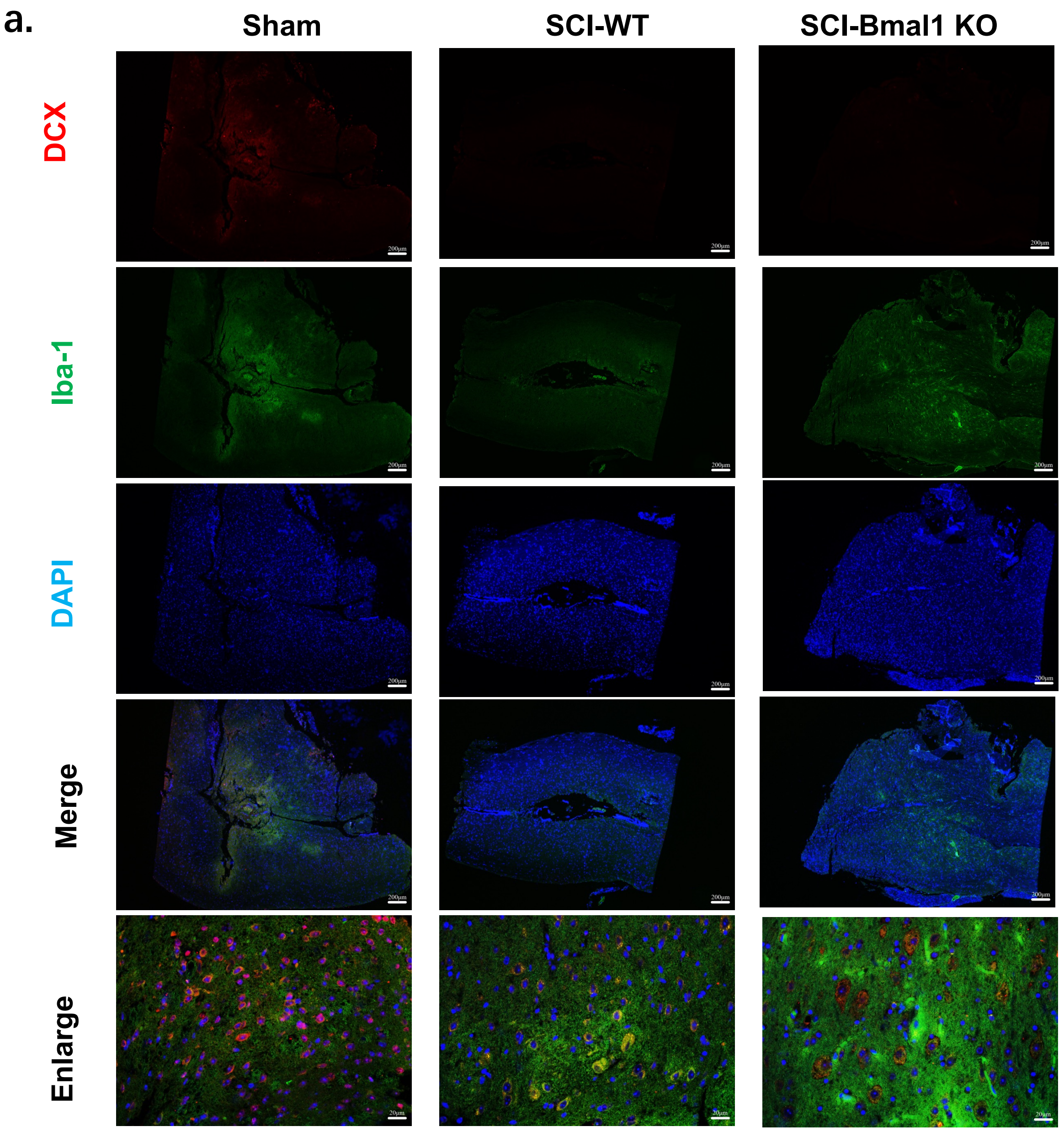

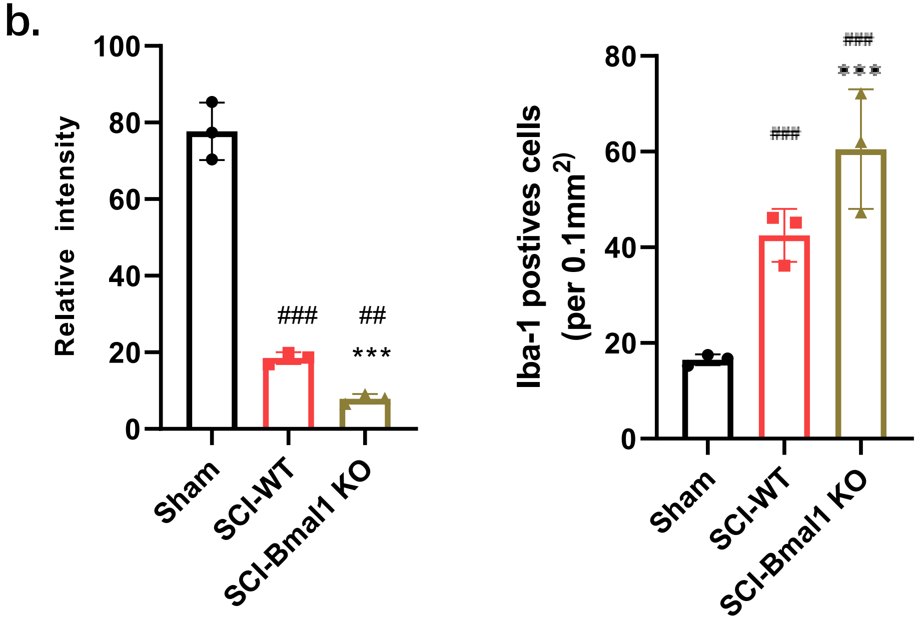

Figure S3. The changes in neuronal and microglial markers following spinal cord injury. (a) Immunofluorescence staining was used to observe the neural-like cells and positive microglia in each group.(b) Quantitation of DCX intensity and Iba-1positive cells.(n = 3 , scale bar = 20 μm. All the data are expressed as means ± SD, one -way ANOVA followed by Tukey's post hoc test was applied; *P<0.05, **P<0.01, ***P<0.001 vs. SCI-WT; # P < 0.05, ##P < 0.01, ###P < 0.001 vs.Sham.)

Figure S4

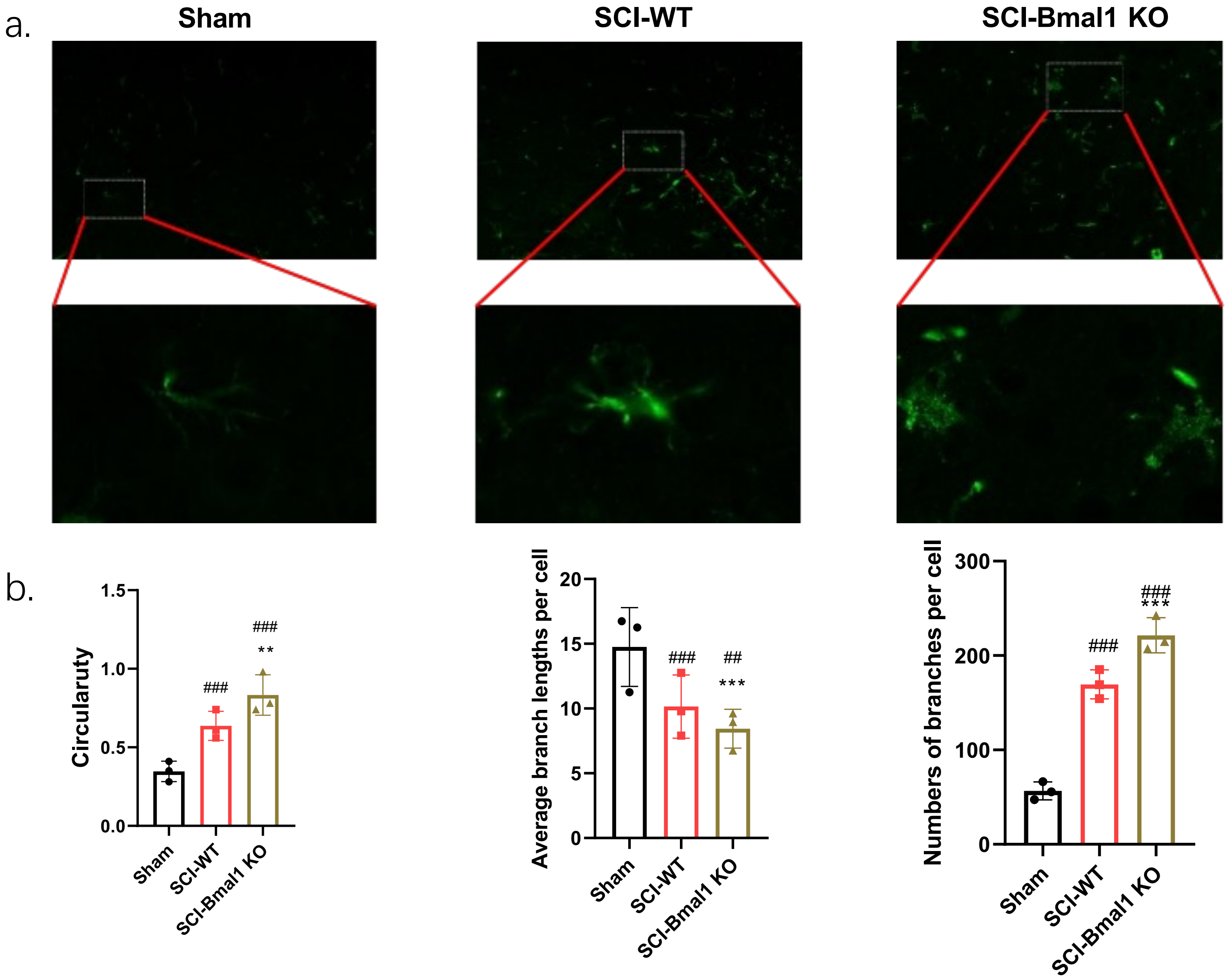

Figure S4. The changes of microglia after spinal cord injury. (a) Immunofluorescence staining was used to observe the morphological changes of microglia in each group.(b) Quantitation of various parameters (circularity, numbers of branches and average branch lengths) for microglia in each group.(n=3, scale bar = 50 μm. All the data are expressed as means ± SD, one -way ANOVA followed by Tukey's post hoc test was applied; *P<0.05, **P<0.01, ***P<0.001 vs. SCI-WT; # P < 0.05, ##P < 0.01, ###P < 0.001 vs.Sham.)

Figure S5

a.

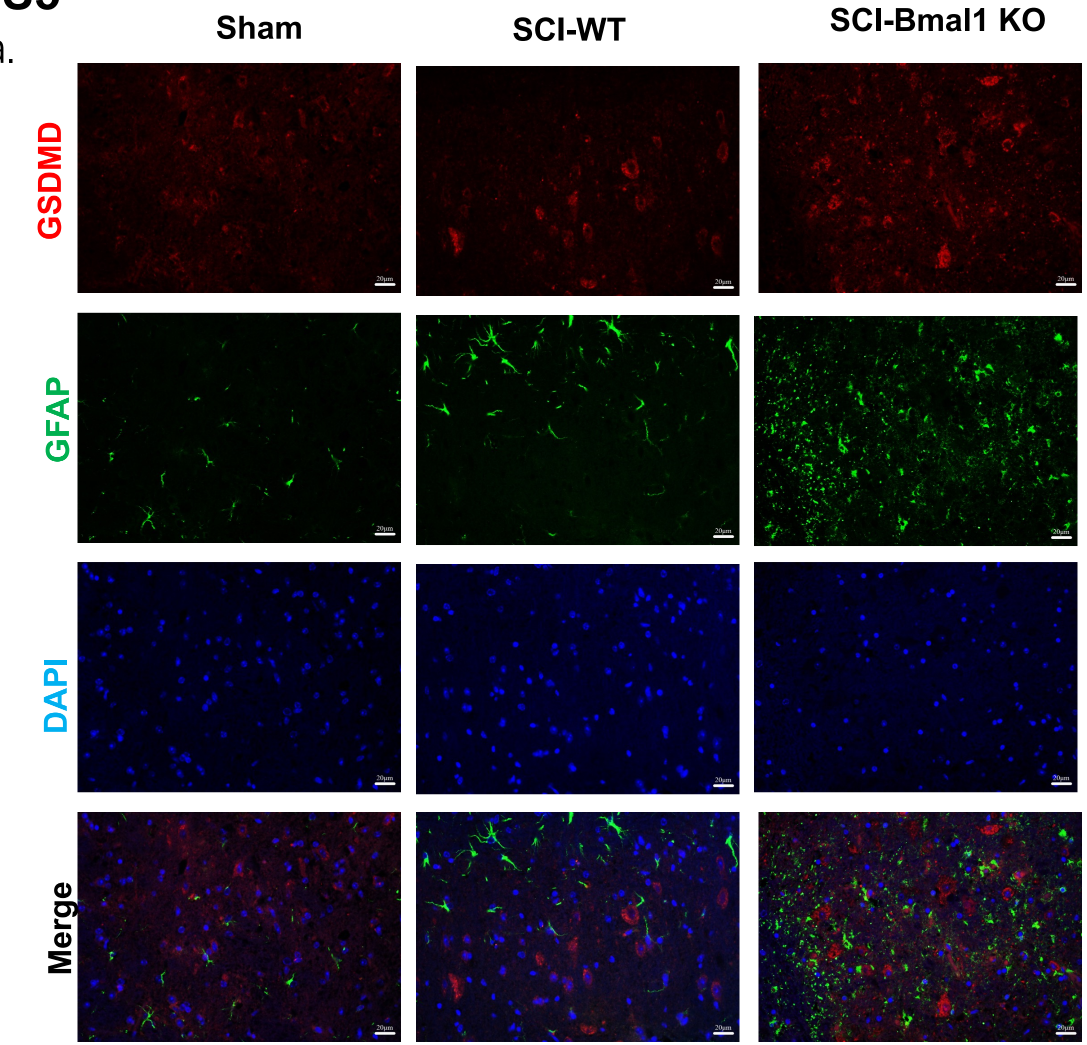

b.

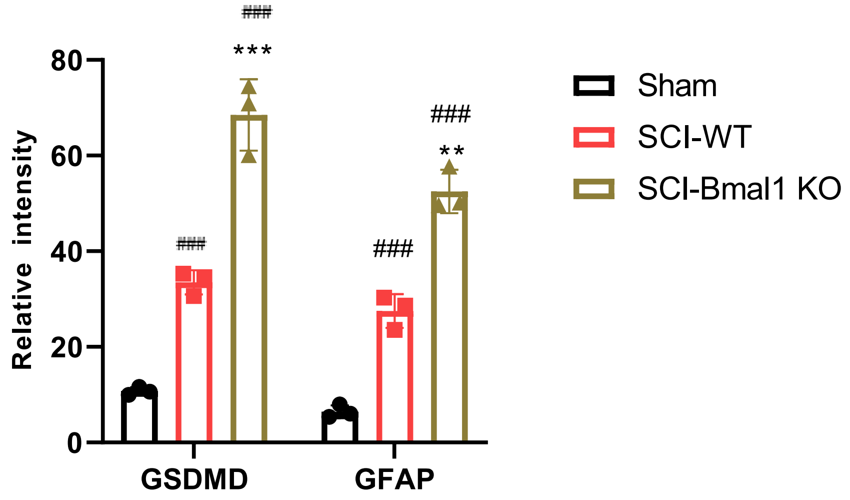

Figure S5 . Representative two-photon excitation images of immunofluorescence of GSDMD and GFAP acquired from WT or Bmal1 KO mice post-spinal cord injury or sham surgery as well as quantitative analysis. (n=3,scale bar = 20 μm, the relative contents of GSDMD and GFAP were calculated using Image J software)。(All the data are expressed as means ± SD, two- way ANOVA followed by Tukey's post hoc test was applied. * p<0.05， ** p<0.01， *** p<0.001 vs.SCI-WT; #P< 0.05, ##P < 0.01, ###P < 0.001 vs.Sham.)

Figure S6

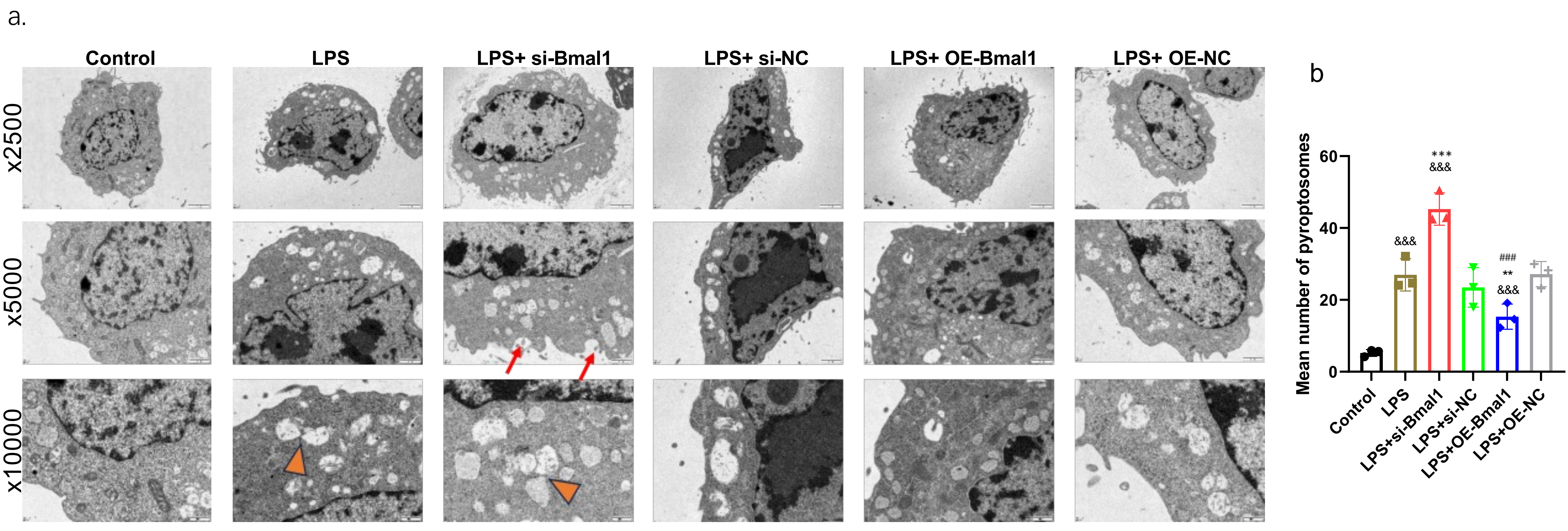

Figure S6. (a) Representative transmission electron microscopic image of ultrastructure of BV2 cells treated with LPS (scale =1μm, scale =2μm, scale = 500 nm, red arrow indicates the perforation and rupture of the cell membrane, orange triangle represents pyroptosome). (b) Quantification of pyroptosomes in representative electron microscopy images. (All the data are expressed as means ± SD, n=3, one - way ANOVA followed by Tukey's post hoc test was applied *p<0.05, **p<0.01, ***p <0.001 vs.LPS; #P < 0.05, ##P < 0.01, ###P < 0.001vs. LPS+si-Bmal1; &P < 0.05, &&P < 0.01, &&&P < 0.001, vs. Control.)

Table S1

BMS score data of each mouse.																		
Days after operation	0 days			3 days			7 days			14 days			21 days			28 days		
Sham	9	9	9	9	9	9	9	9	9	9	9	9	9	9	9	9	9	9
	9	9	9	9	9	9	9	9	9	9	9	9	9	9	9	9	9	9
	9	9	9	9	9	9	9	9	9	9	9	9	9	9	9	9	9	9
	9	9	9	9	9	9	9	9	9	9	9	9	9	9	9	9	9	9
	9	9	9	9	9	9	9	9	9	9	9	9	9	9	9	9	9	9
	9	9	9	9	9	9	9	9	9	9	9	9	9	9	9	9	9	9
	9	9	9	9	9	9	9	9	9	9	9	9	9	9	9	9	9	9
	9	9	9	9	9	9	9	9	9	9	9	9	9	9	9	9	9	9
	9	9	9	9	9	9	9	9	9	9	9	9	9	9	9	9	9	9
	9	9	9	9	9	9	9	9	9	9	9	9	9	9	9	9	9	9
SCI-WT	0	0	0	0	0	0	2	2	2	3	4	2	5	6	4	6	7	5
	0	0	0	0	0	0	2	1	3	2	3	1	2	3	1	3	3	3
	0	0	0	0	0	0	1	1	1	3	3	3	4	4	4	4	4	5
	0	0	0	0	0	0	1	2	1	1	2	1	2	2	2	3	4	3
	0	0	0	0	0	0	2	1	1	2	2	2	3	4	2	3	3	3
	0	0	0	0	0	0	1	1	1	2	2	2	3	3	3	4	4	4
	0	0	0	0	0	0	1	1	1	3	2	4	3	3	3	3	3	3
	0	0	0	0	0	0	3	4	2	1	1	1	2	1	3	4	5	4
	0	0	0	0	0	0	2	1	3	4	3	5	4	4	4	4	5	3
	0	0	0	0	0	0	2	2	2	3	4	2	4	4	4	5	4	6
SCI-Bmal1 KO	0	0	0	0	0	0	0	0	0	0	0	0	1	2	1	1	2	1
	0	0	0	0	0	0	1	1	1	2	1	3	2	2	1	2	2	2
	0	0	0	0	0	0	0	0	0	1	1	2	2	2	2	2	2	2
	0	0	0	0	0	0	1	1	1	2	3	1	2	2	2	2	2	2
	0	0	0	0	0	0	0	0	0	1	1	1	1	1	1	1	2	1
	0	0	0	0	0	0	0	0	0	1	1	1	1	1	2	3	2	3
	0	0	0	0	0	0	0	0	0	1	1	1	1	1	1	2	1	2
	0	0	0	0	0	0	1	1	1	1	2	1	2	3	2	2	2	2
	0	0	0	0	0	0	1	2	1	1	1	1	2	3	2	2	2	2
	0	0	0	0	0	0	0	0	0	0	0	0	1	1	1	3	2	3

Days after operation	Sham	SCI-WT	SCI-Bmal1 KO
0	9	0	0
3	9	0	0
7	9	1.67±0.80	0.43±0.57
14	9	2.43±1.07	1.07±0.78
21	9	3.03±1.13	1.60±0.62
28	9	4.16±1.08	1.97±0.56

Table S1. BMS score data of mice.
